# Binary image representation of a ligand binding site: its application to efficient sampling of a conformational ensemble

**DOI:** 10.1186/1471-2105-11-256

**Published:** 2010-05-18

**Authors:** Edon Sung, Sangsoo Kim, Whanchul Shin

**Affiliations:** 1 Department of Chemistry, Seoul National University, Seoul 151-742, Korea; 2 Department of Bioinformatics, Soongsil University, Seoul 156-743, Korea

## Abstract

**Background:**

Modelling the ligand binding site of a protein is an important component of understanding protein-ligand interactions and is being actively studied. Even if the side chains are restricted to rotamers, a set of commonly-observed low-energy conformations, the exhaustive combinatorial search of ligand binding site conformers is known as NP-hard. Here we propose a new method, ROTAIMAGE, for modelling the plausible conformers for the ligand binding site given a fixed backbone structure.

**Results:**

ROTAIMAGE includes a procedure of selecting ligand binding site residues, exhaustively searching rotameric conformers, clustering them by dissimilarities in pocket shape, and suggesting a representative conformer per cluster. Prior to the clustering, the list of conformers generated by exhaustive search can be reduced by pruning the conformers that have near identical pocket shapes, which is done using simple bit operations. We tested our approach by modelling the active-site inhibitor binding pockets of matrix metalloproteinase-1 and -13. For both cases, analyzing the conformers based on their pocket shapes substantially reduced the 'computational complexity' (10 to 190 fold). The subsequent clustering revealed that the pocket shapes of both proteins could be grouped into approximately 10 distinct clusters. At this level of clustering, the conformational space spanned by the known crystal structures was well covered. Heatmap analysis identified a few bit blocks that combinatorially dictated the clustering pattern. Using this analytical approach, we demonstrated that each of the bit blocks was associated with a specific pocket residue. Identification of residues that influenced the shape of the pocket is an interesting feature unique to the ROTAIMAGE algorithm.

**Conclusions:**

ROTAIMAGE is a novel algorithm that was efficient in exploring the conformational space of the ligand binding site. Its ability to identify 'key' pocket residues also provides further insight into conformational flexibility with specific implications for protein-ligand interactions.

## Background

Computer modelling and simulations plays an important role in the drug discovery process [1]. All protein molecules are inherently dynamic and may have multiple conformers with similar energies in both the bound and unbound states [2-6]. The model of preexisting conformations, together with the "induced-fit" where the ligand binding event causes a change in protein conformation, can explain the process of protein-ligand binding. Nevertheless the experimental static structures obtained by the X-ray and nmr techniques, let alone the modelled structures, are the bases for various structure-based drug design strategies such as de novo design and docking [7-12]. In these efforts, the consideration of the protein flexibility is important [13-16]. Even the conformational change of a single amino acid residue can change the size, shape, and electrostatic property of the binding pocket. Comparisons of the X-ray structures of the pairs of the holo and apo forms indicate that the degree of structural change of the protein varies considerably [17-21]. Some protein undergoes rather large conformational change of the backbone. In the majority of the cases studied, however, the conformational changes occur only at the side chain level.

Here we present a new method, ROTAIMAGE, for modelling the plausible conformers for the ligand binding site given a fixed backbone structure. There are numerous methods to predict the side-chain conformations as a component of protein structure prediction and protein redesign. This is known as the classical side-chain prediction problem [22]. Most of these methods utilize the rotamer library that restricts side-chain torsion angles to a set of preferred or commonly-observed low-energy conformations of a particular side chain [22-26]. Due to its combinatorial nature, it is considered as NP-hard and the number of conformers grows steeply as more residues are considered. For example, 10^5^~10^6 ^bump-free conformers were generated for a set of 11 residues, while the number went up to over 10^10 ^conformers for 19 residues (Table 1). Application of docking to all these conformers, albeit plausible, is not practical. From now on, we call this computational burden due to high number of conformers to handle as 'computational complexity'. Various algorithmic heuristics have been applied in order to reduce the 'computational complexity' such as Monte Carlo simulations [27,28], steepest-descent minimization with random restarts [29], and self consistent mean-field approaches [30]. The tool SCWRL combines the Dead-End Elimination (DEE) algorithm, which restricts the conformational space, with an exhaustive but fast search of the remaining conformational space [23,31,32].

**Table 1:** Statistics on bit string generation for MMP-1 and MMP-13 ligand binding pockets.

Item	MMP-1	MMP-13 full	MMP-13D	MMP-13P
# of FLRs^1^	23	27	23	13

# of SLRs^2^	13	21	13	8

# of CCRs^3^	11	19	11	5

# of all 3D points	809	1,222	614	621

# of points free of contacts	578	740	223	507

# of conditional contact points	231	482	391	114

# of all conformers free of bumping	362,862	71,066,419,200	6,326,207	1,380

# of non-redundant bit strings	8,000	-	358,752	143

# of unique strings after merge^4^	-	-	33,172	-

Reduction in 'computational complexity'^5 ^(f old)	41	-	190.7	9.58

Run time for two-way clustering (Cluster 3.0)^6^	9min		370 min	<1 sec

^1^The residues within 5 Å from the ligand ensemble

^2^The residues within 3 Å from the 3D points that are within 1 Å from FLRs

^3^The residues showing 'conditional' contacts with the 3D points

^4^Bit strings differ by less than 5 bits were merged

^5^The ratio defined as [# of all conformers free of bumping]/[# of non-redundant bit strings]

^6^CPU time elapsed using an Intel Xeon E5430 CPU operating at 2.66 GHz with 24 GB of main memory (the maximum virtual memory usage was 4.4 GB) on a linux box.

An alternative approach, called DYNASITE, has been developed with the aim of reducing the 'computational complexity' by clustering the conformers based on structural similarities and performing docking against a representative conformer of each cluster [25,33,34]. It used principal component analysis (PCA) to cluster the conformers based on *rmsd *between the flexible side chain atoms and successfully generated one of the known conformers starting from a different conformer as a template. However, some practical limitations were noticed with this approach. In order to keep the number of conformers for clustering under a practical limit (~10^4^), the rotamer combination was restricted to the pocket residues with movements observed experimentally; the maximum number of pocket residues was 11 for the proteins studied [35].

Each conformer of the pocket residues generates a unique shape of complementary pocket volume. Thus clustering based on the pocket shape dissimilarity would be equivalent to that based on structural *rmsd *between conformers. Our method presented here, ROTAIMAGE, clusters the pocket conformers based on the dissimilarity of their complementary pocket volumes. The shape similarity between two different volume objects can be defined as a relative volume overlap between them. Since they are already in common coordinate system, the relative volume overlap can be numerically integrated over a set of three-dimensional (3D) grid points that encompass the ensemble of the pocket volumes. The grid spacing controls the granularity of volume assimilation. At its given level, the pocket volumes that differ only in fine detail can be treated as identical.

Computationally, each 3D volume object is distinguished by the status of every one of the 3D grid points whether one is inside or outside the volume. The status of a point is encoded into a bit position of a string, whose value instances a particular pocket surface shape. This is widely used in the field of 3D image recognition in computer science. Representation of a volume object by a bit string enables efficient operation of tasks such as identification of those objects that are treated as having identical shapes at the given granularity and calculation of similarity index between two objects. For example, the former can be achieved by storing the bit strings into a hash table. While preserving the connection between a conformer and a bit string, only unique strings are identified and used in the subsequent analyses. Compared to the original number of conformers, one would expect reduction in the number of unique strings. The reduction in 'computational complexity' was substantial with our test cases, allowing to enumerate sterically allowed conformers with more pocket residues than those used by DYNASITE and to yield a manageable number of unique bit strings for clustering. Calculation of the dissimilarity between two strings, defined as the Tanimoto coefficient between them, involves bitwise AND and OR operations and should be more efficient than that of *rmsd *between two conformers.

Application of standard clustering algorithms using the dissimilarity matrix would segregate the pockets by the similarity in shape. Selection of a representative pocket shape from each cluster would reduce the 'computational complexity' to a degree amenable to the downstream ligand docking in a practical way. For example, each of our test cases yielded a handful of clusters. Here we present details of ROTAIMAGE and demonstrate its utility by modelling the ligand binding site of human matrix metalloproteinase-1 (MMP-1) and -13 (MMP-13), which have been actively studied due to their pharmacological and biochemical importance [25,33,36,37].

## Methods

### Algorithm

**Figure 1:**
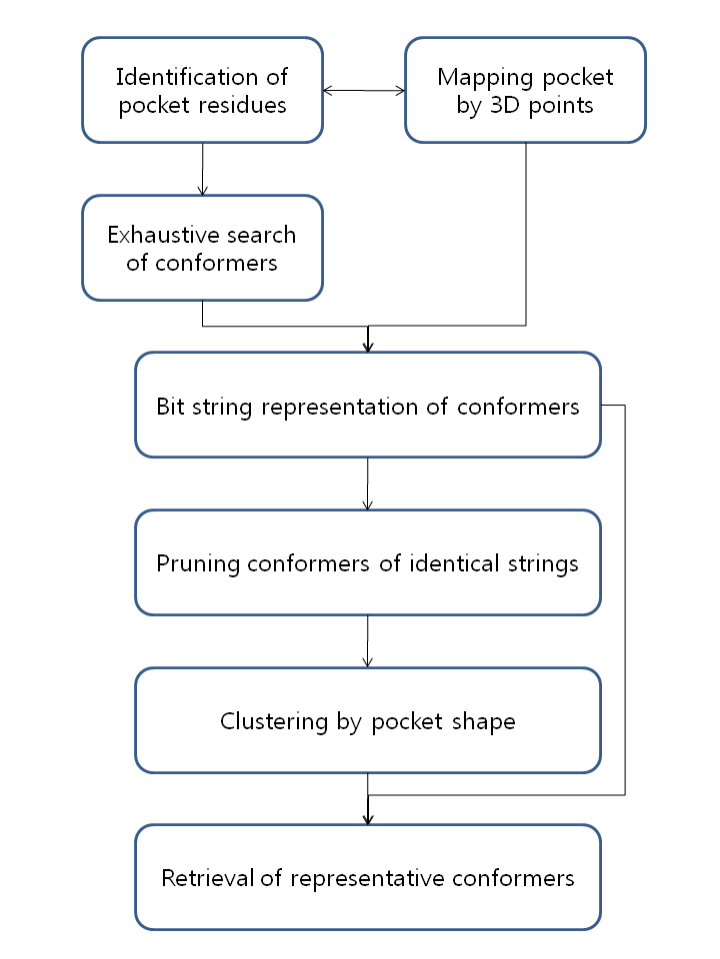
**Outline of the ROTAIMAGE algorithm**. Each box represents an identifiable step of the ROTAIMAGE algorithm. The arrows indicate the flow of information. The top three small boxes constitute the preparatory steps.

The main objective of our algorithm, ROTAIMAGE, is to manage the ligand binding pocket shape using a bit string and to apply this technique to reduce the 'computational complexity' in handling the binding site conformers generated through the exhaustive search of rotameric combination of side chains on fixed backbone. There are three preparatory steps prior to the main algorithm: (i) recognition of binding site residues; (ii) mapping the binding pocket by 3D points; (iii) generation of multiple binding site conformers by rotamer assignment and concurrent checking for steric clashes. The core steps include checking the contact between the 3D points and each conformer and binary representation of the contact configuration, and pruning the conformers that produce identical bit strings. The resulting set of bit strings are then analyzed using standard clustering algorithms. The algorithm is outlined in Figure 1 and described below in detail.

### (1) Recognition of binding site residues and mapping the binding pocket by 3-D points

Identification of ligand binding pockets and analysis of their shapes have been an active area of research, as reviewed by Weisel et al. [38]. There are many algorithms available for this, such as PASS [39], POCKET [40], LIGSITE [41], SURFNET [42], CASTp [43], and PocketPicker [38,44]. Our main objectives are to explore the conformational varieties of the ligand binding site via rotameric combination, and to map the corresponding variations of the pocket shape. For this, we need a maximally expanded pocket where the side chains of the pocket residues are substituted by Ala. Thus the following simple and straightforward approach would suffice for our purpose. For a given protein, the crystal structures of reasonably high resolution are collected. If multiple structures are available, one of them is chosen as a template and all the others are superposed to it. Since our algorithm assumes fixed backbone conformation, any outliers in terms of backbone overlaps are excluded from consideration. Although our algorithm should work with a template structure of either ligand-bound (halo) or ligand-free (apo) form, we describe first the procedure for a halo form as the template. The superposition of protein backbones creates a ligand ensemble if multiple structures of ligand complexes are available. In order to identify the residues that are subject of conformational search, we conduct multiple steps of selection and pruning (see Additional File 1: Figure S1 for the schematic representation). Firstly, all protein residues that are within 5 Å of the ligand ensemble are identified and refer to as the 'first layer residues (FLRs)'. These residues except for Gly are then substituted with Ala. This creates an afore-mentioned artificial volume enlarged compared to the original ligand-binding pocket. It would be the volume maximally accessible by the exploration of side-chain conformations with fixed backbone atoms. Nevertheless, it is referred to as the ligand-binding pocket. The second step is the generation of the 3-dimensional points that map this ligand-binding pocket. As mentioned above there are several algorithms available for this and we use POCKET, which generates a lattice-like 3D array and allows rather simple manipulation of the lattice spacing. The lattice spacing is set to 1.0 Å. The 3D lattice points fill in the pocket by approaching the binding site residues as close as their van der Waals radius. The points that are more than 6 Å away from the binding site residues or ligand ensemble are trimmed. Once side chains are put back into the FLR, they are likely in contact with the outer layer of the 3D points. The last step is the recognition of the residues whose rotameric combination would be explored. If we limit the scope to those residues that might have direct contacts with the 3D points and do not allow simultaneous flexible movement of residues deeper inside the protein core, most rotameric variations except a few would face resistance from the wall of those secondary residues. Hence, it would make more sense to consider the residues underneath the pocket surface than the residues within a certain distance from the ligand ensemble like the FLRs. For convenience, we define the 'second layer residues (SLRs)' as those residues within 3 Å from the 3D points that are in contact with FLRs. Some FLRs are bound to be included in SLRs, but not all of them. Selection of FLRs depends on the ligands included in the ensemble since the ligands are filtered by the distance from the FLRs. On the other hand, selection of the SLRs is not directly influenced by the choice of the ligand ensemble. Rather the shape of the pocket dictates the distribution of the 3D points, which, in turn, governs the SLRs.

If no halo form can serve as the template structure, one would select FLRs manually based on prior knowledge from literature or skip it to the 3D point generation. For the latter case, the potential pocket residues are not substituted by Ala and thus the pocket is not maximally expanded. Once 3D points are generated, SLRs can be identified by the same procedure. However the number of SLRs would be somewhat less than that of the former.

### (2) Rotamer combination and steric clash tests

We take into account the side chain flexibility by placing the so-called backbone-independent rotamers [32] back on each of the SLRs defined in the previous step and performing an exhaustive search of binding site conformers. In general, the computational load for enumerating combinations of rotamers and subsequent filtering of the steric clashes in the resulting conformers grows exponentially with increasing number of rotamers. In order to circumvent this problem, the rotamer library is reduced by checking the bumping between the main chain and the side chain of each rotamer using Equation (1) [25]. Two atoms A1 and A2 are considered to clash if(1)

where CTD stands for clash tolerance distance. Based on the reduced library of the surviving rotamers only, the interactions between the residues are mapped as a graph, which is then searched by a backtracking and branch-and-bound algorithm that concurrently checks the bumping between the side chain atoms of each pair of residues. This part of our algorithm follows the published graph theoretic cluster algorithm in SCWRL [24], except for the step of dead-end elimination and the scoring functions themselves.

### (3) Binary image representation in protein binding site

**Figure 2:**
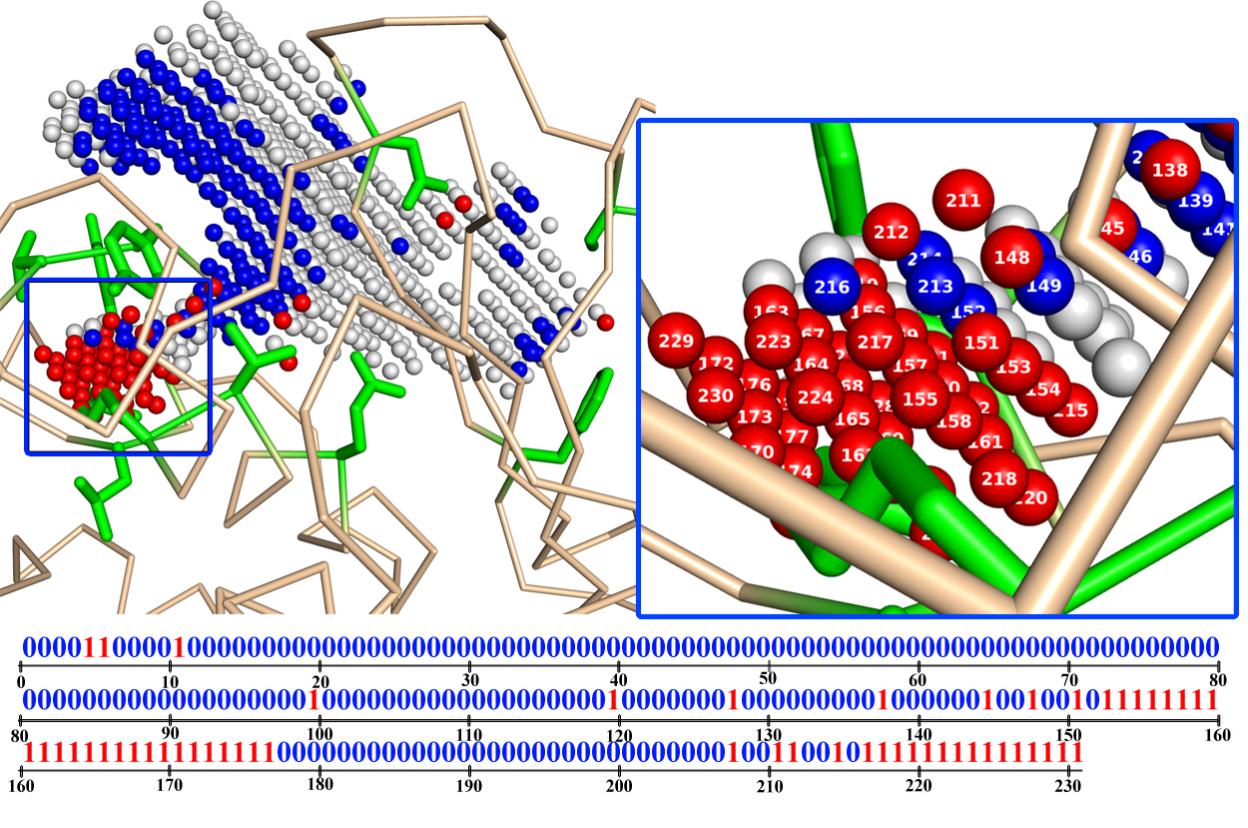
**The ligand binding pocket of MMP-1 for an instance of a conformer**. The pocket enlarged by Ala-substitution was mapped by 809 3D points using the POCKET algorithm. The 3D points are depicted by small spheres color-coded according to their contact states: 578 points were free of contacts with any of the conformers (gray); 231 points that had 'conditional' contacts with some but not all of the conformers were shown in blue (free of contact for a given conformer) or red (in contact in this case). In the zoomed portion, the points are labeled and their contact status is coded at the corresponding position in the bit string shown below (1 in contact; 0 for free of contact).

The 3-dimensional points representing the enlarged binding pocket as obtained in the first step are screened for overlap with the atoms of each conformer that has been generated by rotamer combination and that has survived the subsequent bumping checks. The contact between a binding site point 'P' and a SLR atom 'A' is evaluated using(2)

where CPD stands for contact padding distance, which is set to 0.5 Å. Summarizing the results over all the conformers, the points always in contact with some SLRs regardless of conformers are eliminated, while the points always free of contacts are set aside and included later in the complete construction of the binding site. Only those points in 'conditional' contacts depending on the conformers are retained to discriminate the conformers. For some residues in the SLRs, none of the rotamers may contact the 'conditional' points at all; these SLRs are eliminated. The rest of the SLRs, which have 'conditional' contacts with the 3D points, have potential for modulating the pocket shape and are defined as 'conditional contact residues (CCRs)'. As shown in Figure 2, each conformer is represented by a configuration of which points are in contacts or not. As it is a combination of binary states, the result is conveniently encoded by a bit string where each bit designates whether the corresponding point is in contact or not. Such representation of a 3-dimensional object by a bit string has been widely used in the field of image recognition in computer science [45].

### (4) Pruning multiple conformers having identical bit strings

The mapping relationship between the bit strings and the conformers is stored in a hash table. By looking up those strings that are observed more than once, we can identify the strings that are mapped to multiple conformers. These conformers have the identical status of contacts with the 3D points, i.e., their pocket shapes are indistinguishable at the specified CTD in Equation (1). There are several situations when this can happen. As described in the previous section, some SLRs are not included in the CCR list. Their rotamers would not generate new bit strings. Even for CCRs, some of their rotamers may not contact the 3D points, resulting in some redundant strings. It is likely to occur at the periphery of the pocket. Besides, the coarser 3D points may cause the less sensitive differentiation of conformers. In such cases identical bit strings may also result from different conformers. Since we keep track of which conformers are assigned to which bit strings, we do not lose any detail and reduce the computation complexity by dealing with non-redundant bit strings in the subsequent process.

### (5) Clustering analysis of multiple binding sites and selection of representative conformers

The shape of the binding site is approximated using a set of 3D points, which are recorded as simple bit strings of length equal to the number of points. Each bit position corresponds to a sphere centered at a defined 3D point. A shape-fingerprint Tanimoto (SFT) coefficient between two such bit strings [46,47], given by(3)

measures the proportion of bits shared between the two strings, where *N*_*A *_and *N*_*B *_represent the number of bits set in the strings *A *and *B*, respectively, while *N*_*A B *_is the number of bit position set commonly in A and B. This is equivalent to a finite numerical integration of the relative volume overlap between two 3D volume objects. A relative volume overlap between two 3D objects can be considered as one of the similarity indices, whose approximation can be given by a Tanimoto coefficient. With such a similarity index, clustering analysis such as multidimensional scaling (MDS) can be performed. The optimum member of clusters is determined through a visual inspection of the MDS plot in 3D using GGobi [48]. MDS was conducted using the R statistical system [49]. A two-way hierarchical clustering of both bit strings and bits can be useful in analyzing the dependency between conformers and 3D points. It was done with the Linux implementation of Cluster 3.0 [50] using an *m *× *n *bit pattern matrix, where *m *and *n *are the number of unique bit strings and the bit size, respectively. The time complexity of complete linkage hierarchical clustering is known as *O*(*N*^2^log*N*) [51]. It should be noted that hierarchical clustering involving a few tens of thousands of bit strings can take extremely long (see Table 1).

One would need a representative conformer for each cluster for follow-up applications such as virtual screening or structural analyses. Since we use bit strings, a numerical averaging would not make sense. We choose the medoid string as the representative of each cluster. The medoid is defined as the member of the cluster whose average dissimilarity to all the other members is minimal [52]. The pocket shapes and conformers corresponding to the medoid strings can be retrieved from the stored mapping information (see the Section (4) of Methods).

## Results and Discussion

### Target proteins

We have tested our algorithm, ROTAIMAGE, by modelling the ligand-binding pockets of MMP-1 and MMP-13. There are several reasons why we have tested our algorithm using these two MMPs: (1) they are important targets for drug design and many inhibitors have been developed [53,54]; (2) multiple ligand-bound complex crystal structures have been determined for each of them at reasonably high resolution [36,37,55-58]; (3) most importantly, their crystal structures showed multiple conformer configurations in the ligand binding pocket [25,36,37]. In addition, their conformational differences are large enough to prohibit rigid-body docking simulations of a known ligand from one configuration to the crystal structures from another configuration. It would be interesting to see whether we can recover all of the conformers seen in the crystal structures from its clustered pools of conformers, or more precisely, whether those ligands could be fit into the representative binding pocket shapes that would result from the clustering of a number of shapes. It would be more important to know how many clusters we have to consider in order to cover all the experimentally observed ligands. Beyond the commonalities, the ligand binding sites of MMP-1 and MMP-13 have quite distinct shapes and properties. The side chain of Arg 214 of MMP-1 limits the S1' pocket to a size suitable for an aromatic ring, while the corresponding residue in MMP-13 is replaced by Leu 218 [36], creating a long channel called S1'* [37]. Consequently, many more residues are involved in MMP-13 than MMP-1. By testing our algorithm with cases of different complexities, we may gain more insight on variations of its performance.

### Generation of multiple binding site images

We downloaded eight and seven crystal structures determined at a resolution of 2.5 Å or higher for MMP-1 and MMP-13, respectively, from Protein Data Bank (PDB) [59]. Each set of proteins were superposed into a common reference structure (966c (1.7 Å resolution) and 1xuc (1.9 Å resolution) for MMP-1 and MMP-13, respectively). One of the crystal structures of MMP-1 displayed a backbone structure distinct from the rest and was excluded in the subsequent analysis. Otherwise the average *rmsd *among backbone Cα atoms were 0.343 Å and 0.328 Å for MMP-1 and MMP-13, respectively.

The residues within 5 Å from the ligand ensemble (FLRs) were identified and substituted with Ala. The enlarged pockets were then filled with 3D lattice points of 1 Å spacing. Since FLRs are determined based on the distances from the known ligands, they may reflect the current repertoire of ligands. In order to remove such a bias and use residues that are within a constant depth underneath the pocket, we looked for residues within 3 Å from the 3D points. These residues are referred to as SLRs. See Figure 1, for the definition of FLRs and SLRs. Some FLRs deep inside the protein core were not used as SLRs, while new ones were added at the periphery where the known ligands did not reach (Figure 1). The next step involved an exhaustive search of conformers through rotamer combination and a concurrent bumping check. In order to tolerate some uncertainty in the atomic positions, we also adopted the clash tolerance schemes defined in Equation (1), where CTD was set at 1.0 Å [35]. For each conformer, the 3D points that were in contact with the side chain atoms were screened using Equation (2) by setting CPD and A to 0.5 Å and van der Waals radius, respectively. After scanning all the conformers, the points were classified into two groups. When this scheme was applied to MMP-1, 11 residues out of the FLRs were retained as CCRs (Table 1). In contrast, a smaller reduction occurred with MMP-13, i.e., from 27 FLRs to 19. The number of 3D points indicated that the ligand-binding pocket of MMP-13 was ~50% larger than the binding pocket of MMP-1 (1,222 vs 809). After excluding those points free of contact with any of the conformers, there were twice as many 3D points with conditional contacts in MMP-13 than in MMP-1 (482 vs 231). The number of CCRs or 3D points showing conditional contacts is correlated to the size of the pocket surface area, not directly to the volume. These numbers were in huge excess of the volume increase in MMP-13 compared to MMP-1, indicating a larger ratio of contact surface-to-volume in the former than in the latter. This may be explained by the fact that MMP-13 had a long and extended binding pocket unlike MMP-1, which had a globular ligand-binding site [36].

The 'computational complexity' due to a large number of conformers is governed by the number of CCRs, while the number of 3D points showing conditional contacts with CCRs dictates the variety of pocket shapes. In this regards, MMP-1 and -13 were useful examples in assessing the complexity associated with these variables. While the exhaustive rotameric combination generated 362,862 sterically allowed conformers for MMP-1, the same procedure produced over 71 billion conformers for MMP-13. Since the number of conformers for MMP-13 was too large to be handled on a reasonable time scale, we decided to split the number of 3D points of MMP-13 into half, with an overlap of points filling the S1' pocket. The "proximal" ligand-binding site that limited to the S1' pocket is called MMP-13P, while the "distal" region that extends to S1'* is called MMP-13D (see for definition Additional File 1: Figure S2). All subsequent analyses were conducted independently. After independently generating the final representative pocket shapes, the full pocket shapes may be produced by combining them based on the status of the 3D points shared between MMP-13D and MMP-13P. Almost twice as many residues were involved in the pocket of MMP-13D than MMP-13P, resulting in huge differences in the sterically allowed conformers (6,326,207 vs 1,380). Interestingly the pocket volumes were similar in size. However, there were 3.4 times more conditional contact points in MMP-13D than in MMP-13P (391 vs 114). This implied that there was a difference in geometric topologies between these two partitions. Overall, we used three test cases of different complexities: MMP-1 (middle level of complexity), MMP-13D (highest level of complexity), and MMP-13P (lowest level of complexity).

The contact status of each point was checked against each conformer and coded into a bit string. Since we used a finite number of 3D points with a fixed non-negligible spacing (1 Å) and the contact check also employed a tolerance (CPD of 0.5 Å in Equation 2), it was likely that some conformers generated identical bit strings. This is one of the interesting features of our approach; for a given 'resolution' defined by the lattice spacing and CPD, we can set a threshold below which minute differences in the pocket shapes can be ignored. This has the effect of greatly reducing the 'computational complexity' without degrading the 'resolution'. For MMP-1, we obtained 8,000 unique strings of 231 bits from a host of 362,862 conformers, reaching at a 41-fold reduction in complexity. MMP-13P had a rather small number of conformers to start with (1,380) and yielded only 143 unique strings of 114 bits (~10-fold reduction). For MMP-13D there were 358,752 non-redundant strings after pruning identical ones. Although more than a 17-fold reduction was achieved, the number of strings was still too high. An additional 10-fold reduction was obtained by merging the strings that differed from each other by less than 5 bits; thus, a total of 33,172 strings were retained (a total of 190-fold reduction). Even with this much of reduction, the two-way hierarchical clustering took more than 6 hours for MMP-13D, while the smaller task with MMP-1 took only 9 min (Table 1).

### Clustering analysis of multiple binding sites

**Figure 3:**
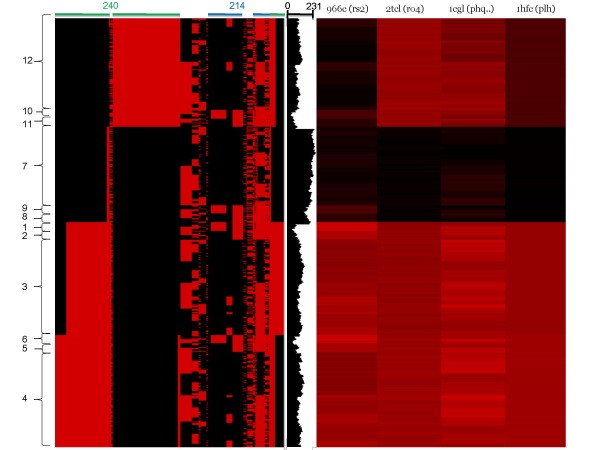
**The heat maps depicting binary image representations of the ligand binding pocket shapes for MMP-1**. On the left panel, each column represents the conditional bits and each row represents the pocket shape of a given conformer. The bit position is set (red) if the conformer is in contact with the 3D points. The bit positions were clustered using an agglomerative complete linkage method, while the conformers were ordered according to the membership in the accompanying MDS plot. Tyr 240 (green) and Arg 214 (blue), the most influential residues in shaping the pockets, are labeled "240" and "214", respectively, over the bit blocks they are in contact with. For each pocket shape, the number of bits off is counted and plotted in the middle panel. On the right panel, the heat map depicts shape incompatibility between a conformer and a ligand. The columns represent the ligands from the known crystal structures, and their incompatibility with each conformer is coded by red (the darker, the more compatible). For example, the clusters 1~6 could not accommodate any of the known ligands.

We have shown that the 'computational complexity' of dealing with many conformers generated by combinatorial rotamer assignment was greatly reduced by representing the conformers as bit strings. Since these bit strings mimic the shape of the binding pocket volume, clustering them would be useful to assess the diversity of their shapes. If a small number of distinctive clusters can be observed, then the entire conformational space accessible by the ligand-binding pocket residues can be encompassed by these clusters and the associated 'computational complexity' can be even further dramatically reduced. Using the non-redundant set of bit strings (8,000, 33,172, and 143 for MMP-1, MMP-13D, and MMP-13P, respectively), a pairwise dissimilarity was calculated as 1 - SFT (Equation (3)). Note that the sizes of the bit strings were 231, 391, and 114 bits for MMP-1, MMP-13D, and MMP-13P, respectively. Based on the resulting shape dissimilarity matrix, multidimensional scaling was conducted (see Additional File 1: Supplementary Figures 3 and 4). Inspection of the principal component plot showed a pattern of distinctive clustering in all three cases, and led us to select 12, 8, and 8 clusters for MMP-1, MMP-13D, and MMP-13P, respectively. After ordering the bit strings according to cluster membership obtained from the MDS analysis, the bit positions were then hierarchically clustered based on their dissimilarity over the conformers. The resulting heat maps are shown in Figure 3 (MMP-1), Additional File 1: Supplementary Figures 5 (MMP-13D) and 6 (MMP-13P). Indeed the conformers belonging to different clusters showed quite distinct bit patterns and interestingly some sets of bit positions that dominated the clustering process were recognized. For example, the bit status of the blocks labeled "240" in Figure 3 combinatorially split the conformers into four super-clusters (1-3, 4-6, 7-9, and 10-12) of MMP-1. Since the information regarding the contact relationship between 3D points and the residues per conformer has been stored, we are able to determine which residues correspond to the bit blocks and consequently are the most influential (vide infra). If the clustering process were to reflect the real dissimilarity in the pocket shape of the conformers, its result should satisfy the following criteria: (1) the atomic *rmsd *within clusters should be less than that between clusters; (2) the ligand-binding conformers observed in the known crystal structures should be found among the non-redundant set of conformers; (3) the conformers similar in their conformation should be found in the same cluster and the ones with distinctive conformations should be in different clusters; (4) the fitness or shape complementarity between a ligand and a conformer should be congruent with the clustering pattern. These points are addressed below.

**Figure 4:**
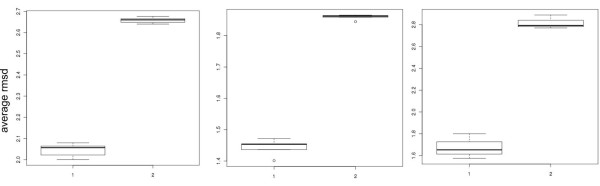
**The box plot of average *rmsd *within and between clusters of MMP-1 (A), MMP-13D (B), and MMP-13P (C)**. From each cluster, the medoid conformer was selected and the structural *rmsd *between the medoid and the rest of the group members was calculated (median at (A) 2.05 Å, (B) 1.45 Å, (C) 1.63 Å). Similarly the *rmsd *between each medoid and the members of the other groups was also calculated (median at (A) 2.65 Å, (B) 1.87 Å, (C) 2.78 Å).

**Figure 5:**
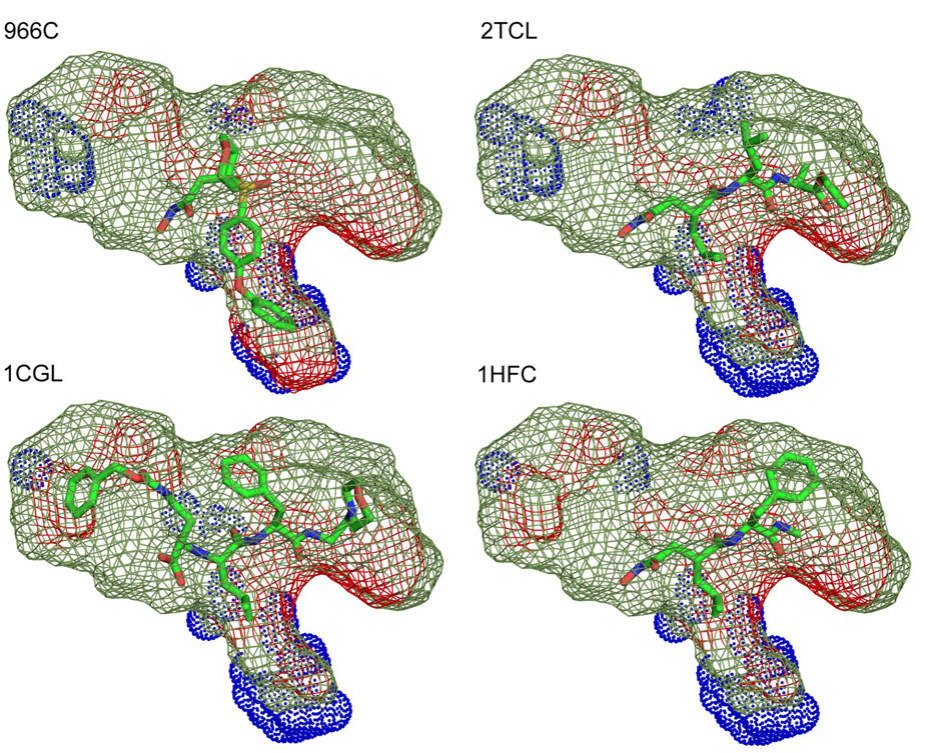
**The pocket shapes of the known ligand-bound crystal structures in MMP-1**. The pocket shapes of the four known conformers were compared visually by depicting the pocket points with the following coloring scheme: the 578 points always free of contacts were in green, while the 231 conditional points were either in red (free of contacts) or in blue (in contacts). Only in 966c was the pocket deep (shown in red) enough to accommodate rs2. In the other three structures, the pocket residues occupied this region (shown in blue).

**Figure 6:**
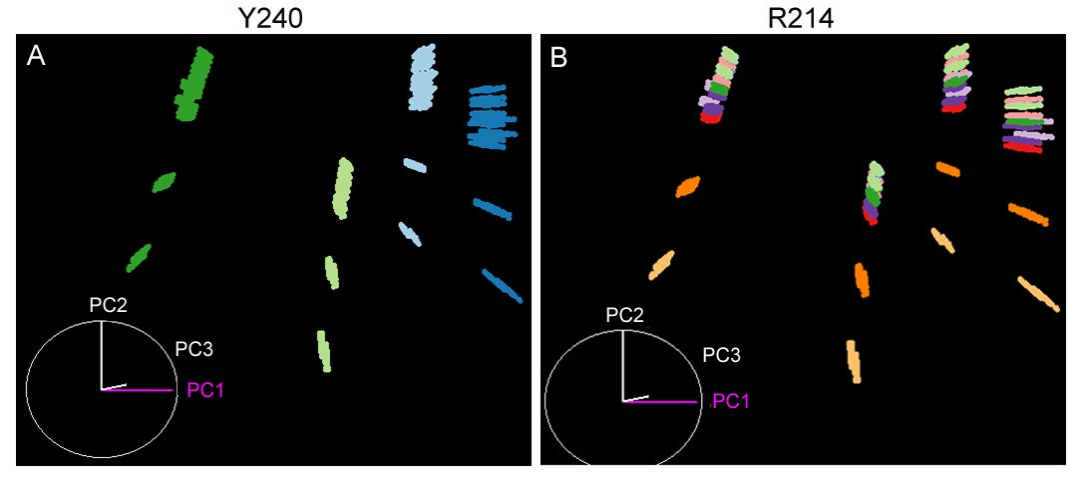
**Rotameric status of the key residues in MMP-1**. A total of 8000 pocket shapes were plotted in the MDS plot (PC1~3). Each conformer is colored by the rotameric status of (A) Tyr 240 and (B) Arg 214.

### Structural dissimilarity of shape clustering

Clustering of the conformers in the previous studies has been based on the dissimilarity in atomic coordinates [25,33-35]. These results are not necessarily identical to those of clustering by pocket shape dissimilarity. On the other hand, if the latter yields irrelevant outcomes compared to those from the former, it would be hard to accept these results. We calculated the *rmsd *between the flexible atoms of the binding site side-chains. The results are presented as boxplots for MMP-1, MMP-13D, and MMP-13P (Figure 4). In all three cases, the atomic *rmsd*'s within a cluster were consistently less than those between clusters. This indicates that the clustering by pocket shape is structurally sound, despite the conceptual difference from the conventional *rmsd*-based clustering.

### Coverage of the conformational space

We have performed an exhaustive search of the conformational space and retained all the sterically allowed conformers in the subsequent bit string representation step. Consequently all the conformers observed in the ligand-bound or apo crystal structures should be mapped into the catalog of the predicted conformers, as long as the known structures did not involve substantial backbone movements. Furthermore, it is likely that some part of the predicted conformational space may not be covered by the known conformers. We, then, asked the following questions: how many of the clusters corresponded to the observed conformers (called "known" clusters) and were there any clusters distinct from the known conformers (called "unknown" clusters). In order to map each known conformer to one of the predicted ones, the closest predicted rotamer was assigned for each binding site residue (SLR) and their combination was examined among the pool of the predicted ones. The results are summarized in Table 2. For MMP-1, all the seven known conformers were mapped into two so-called "known" clusters (one ligand complex structure in the cluster 7, and the other six in the cluster 9), while the other ten clusters have not been observed in the crystal structures. Among the seven known structures, four were in ligand-bound forms (one in the cluster 7, and the other three in the cluster 9). In the heat map showing the contact status of the CCRs with the 3D points (Figure 3), the more bit positions that were set, the less the pocket volume. Compared to the "known" clusters, the "unknown" clusters showed diminished pocket volumes. Their pocket shapes were examined visually (see Additional File 1: Figure S7). It appears that the "unknown" clusters exhibit a somewhat collapsed ligand-binding pocket that is also distinct from the apo form. On the other hand, the known conformers belonged to the clusters that were the widest open as far as CCRs were concerned. The collapsed pocket without the concomitant adjustment of the backbone atoms may result in energetically unfavorable loose packing of the side chain atoms. We may heuristically conclude that a cluster having severely diminished pocket volume is unrealistic. The pocket shapes of the four known ligand-bound crystal structures are shown in Figure 5. The most distinctive difference between the two "known" clusters (7 and 9) was in the region that protruded downward. Only in 966c (Figure 5A) was the pocket deep (shown in red) enough to accommodate rs2. In the other three structures (Figure 5B-D), the pocket residues occupied this region (shown in blue).

**Table 2:** Mapping the known conformers taken from crystal structures to the pocket shape clusters.

protein	pdb code	ligand	cluster ID	
MMP-1	966C	RS2	7	
		
	2TCL	RO4	9	
			
	1CGL	PHQ		
			
	1HFC	PLH		
			
	1CGF	*apo*		
			
	1CGE	*apo*		
			
	2J0T	*apo*		

protein	pdb code	ligand	cluster ID^1^	cluster ID^2^

MMP-13	1XUC	PB3	5	6
			
	1XUD	PB4		
			
	1XUR	PB5		
		
	2OW9	SP6	6	
		
	1ZTQ	O33	3	
			
	830C	RS1		
		
	2E2D	*apo*	4	

^1^MMP-13D

^2^MMP-13P

We have identified eight clusters for MMP-13D from the MDS plot. PC1 separated the four "known" (3~6) and the other four "unknown" clusters (1, 2, 7, and 8). Similar to MMP-1, the "known" clusters displayed bigger pockets than the "unknown" ones. Within the "known" cluster group, the clusters 3 and 4 were separated from the clusters 5 and 6 by PC2 (see Additional File 1: Figure S5). Among the eight clusters identified from the MDS plot for MMP-13P, the cluster 6 had the largest volume and included all the seven known conformers (see Additional File 1: Figure S6).

It should be noted that the "known" conformers shown in Table 2 included both ligand-bound (halo) and ligand-free (apo) forms. In fact all the halo forms except one belonged to the same cluster as the apo forms in MMP-1. On the other hand, the apo form belonged to a cluster distinct from those of halo forms in MMP-13D. As long as the difference between apo and halo forms is in side chain conformation with fixed backbone, we can use one form as the template and can recover the others among the clusters.

### Shape clustering and ligand incompatibility

Having established that the known pocket conformers of similar shapes fall into the same cluster, we next determined whether these clusters can distinguish different ligands or not. The ligand binding pocket of a conformer may easily accommodate some ligands, but not others. If we measure such shape incompatibility between a conformer and a ligand, its profile over a series of ligands should show a uniform pattern for all the member conformers in a given cluster. We define the shape incompatibility between a ligand and a pocket as the volume of the ligand sticking out of the volume encompassed by the pocket. We applied the following algorithm to estimate it. We calculate the distance from every ligand atom to the pocket grid points we have generated based on the template structure. The grid points that satisfy Equation (2) are marked as belonging to the ligand, constituting the volume occupied by the ligand. Since they are mapped on the same lattice, their shapes can be easily compared using bit strings. Let the bit strings describing the ligand volume and the pocket L and P, respectively. The incompatibility is then calculated as L \ P, the relative complement of P with respect to L.

As shown in the heat map (Figure 3 right panel), all members of a cluster had very similar profile of shape incompatibility with the known ligands of MMP-1. The "unknown" clusters 1~6 displayed closed pockets that could not accommodate any of the known ligands. Although the conformers in the clusters 10~12 have not been observed in the known crystal structures ("unknown"), it appeared that their shapes were compatible with that of rs2. Since the relatively small rs2 binds deep inside the pocket, there was little discrimination against the "unknown" clusters. The "known" clusters 7 and 9 were compatible with their respective known ligands. The "unknown" cluster 8 showed an incompatible profile similar to those of the clusters 7 and 9 as the former had a pocket shape similar to those of latter (see Additional File 1: Figure S7). Similar heat maps were plotted for MMP-13D and MMP-13P, separately (see Additional File 1: Supplementary Figures 5 and 6). The two groups of the "known" clusters of MMP-13D separated by PC2 showed different preferences for the known ligands. Our analysis of the bit patterns showed the variation in PC2 was modulated by the rotameric status of Leu 218 (see Additional File 1: Supplementary Figures 5 and 8). Apparently, the wide-open pocket in the clusters 5 and 6 can accommodate all the known ligands, while the narrow-necked pocket in the clusters 3 and 4 preferred o33 and rs1 (see Additional File 1: Figure S9). In MMP-13P, cluster 6 showed very similar patterns of shape incompatibility over the six ligands (see Additional File 1: Figure S6).

### Feasibility of the representative conformers

We have shown that the conformers of the ligand-binding residues were clustered based on their pocket shapes and the clustering pattern reflected the shape incompatibility between a conformer and a ligand. For follow-up applications such as virtual screening or structural analyses, one would need a representative conformer for each cluster. Since the shape incompatibility profile for the known ligands was uniform over a cluster, any member of the cluster could be used to represent the entire cluster. We chose the medoid string as the representative of each cluster (see the Section (5) of Methods). Since the medoid is one of the members, the pocket shape and conformer corresponding to the medoid string can be retrieved from the stored mapping. See Additional File 1: Supplementary Table 1 for the shape incompatibility between the medoid pockets and the known ligands. In MMP-1, all the known ligands including rs2 showed minimal bumping with the medoid of the cluster 7, to which its ostensible binding site conformer mapped. The medoid of cluster 9 was also compatible with the three known ligands excluding rs2, as expected. The compatibility patterns between the cluster medoids and the corresponding known ligands of MMP-13 were consistent with the cluster mapping results.

### Identification of key pocket residues

The two-way clustering heat map of the bit patterns depicting the contacts between 3D points and the pocket residues (Figure 3 and see Additional File 1: Supplementary Figures 5 and 6) implied that some sets of clustered bits were more influential than others in clustering the conformers. If those influential bits were in contact with a few residues only, the latter may be considered the key pocket residues. In order to identify such residues, we ran through the residue list and colored the conformers in the MDS plot by the residue rotamer status. We visually inspected each colored plot to see whether the coloring pattern was segregated along the major principal component (PC) axes. As shown in Figure 6 for MMP-1, the variances along PC1 and PC2 can be explained by the rotameric status of the Tyr 240 and Arg 214 residues, respectively. Similarly, the key residues of MMP-13D were identified as Phe 252, Leu 218, and Lys 249, while those of MMP-13P were Tyr 244, Glu 223, and Ile 243 (see Additional File 1: Supplementary Figures 8 and 10). It should be noted that the ability to discern the key binding site residue is an advantage of the ROTAIMAGE algorithm, which maps the binding pocket by 3D points. Other approaches that are based only on energy calculations of the binding site residues would pinpoint which residue contribute the most to the overall energetics of the conformer, but would not be able to define which residue most affect the shape of the binding pocket. Calculations of the accessible surface area [60] may provide similar information, but it is not necessarily true that more exposed residues influence the binding pocket shape more.

## Conclusions

The ROTAIMAGE algorithm thoroughly surveys the sterically allowed conformational space and reduces the conformational complexity by partitioning the conformers into a reasonable number of groups, as previously proposed [25,33-35]. These approaches are different from the traditional ones, which attempt to search for the global minimum-energy conformation (GMEC) through energy minimization of the conformers. Instead we propose a method to extract a list of representative conformers from the exhaustive set of pocket conformers. Since each one is nearby a distinct local minimum, GMEC can be sought later by exploring the representative conformers in parallel flexible ligand docking exercises. On the other hand, ROTAIMAGE is novel and distinct from DYNASITE in that the former compares and clusters the shapes of the pocket as opposed to the latter that handles the binding site conformers (see Additional File 2 Supplementary Note for a detailed comparison with DYNASITE). Considering that a ligand should fit in the pocket volume, focusing on the shape of the pocket may provide a strong filter for ligand screening. The ROTAIMAGE algorithm creates a 3D shape using a bit string, which is widely used in the field of image recognition in computer science, and is an efficient way to allow easy manipulation of the conformers. Judiciary application of the standard clustering technologies to the bit patterns can partition the conformers into groups that are structurally sensible. Bit representation offers the ability to examine the conformation context of the rotamers in that the most influential residues are determined from analysis of the bit pattern. ROTAIMAGE may find wide acceptance and use in the area of ligand binding pocket modelling.

## Authors' contributions

ES carried out the computational analysis to test the ROTAIMAGE algorithm, helped with the analysis of the results and helped to draft the paper. SK helped with the design of the study, analysed the results, and drafted the paper. WS helped with the design of the study and helped to draft the paper. All authors read and approved the final manuscript.

## Supplementary Material

Additional File 1 **Supplementary Figures and Table**. This file contains 10 additional figures illustrating the results for the MDS scatterplots, heat maps, and rotameric status. Furthermore a supplementary table is shown for shape incompatibility in MMP1, MMP-13D, and MMP-13P. Click here for file

Additional File 2 **Supplementary Note**. This file contains additional description of comparative features of our ROTAIMAGE and DYNASITE or Kalblad et al. [25]. Click here for file
