# Supplementary material for: Binary image representation of a ligand binding site: its application to efficient sampling of a conformational ensemble
Source: BMC Bioinformatics. 2010 May 18;11:256. doi: 10.1186/1471-2105-11-256 (PMC3098062; doi:10.1186/1471-2105-11-256)
Supplement: Additional File 1 — Supplementary Figures and Table. This file contains 10 additional figures illustrating the results for the MDS scatterplots, heat maps, and rotameric status. Furthermore a supplementary table is shown for shape incompatibility in MMP1, MMP-13D, and MMP-13P. [file 1471-2105-11-256-S1.PPT]

## Slide 1
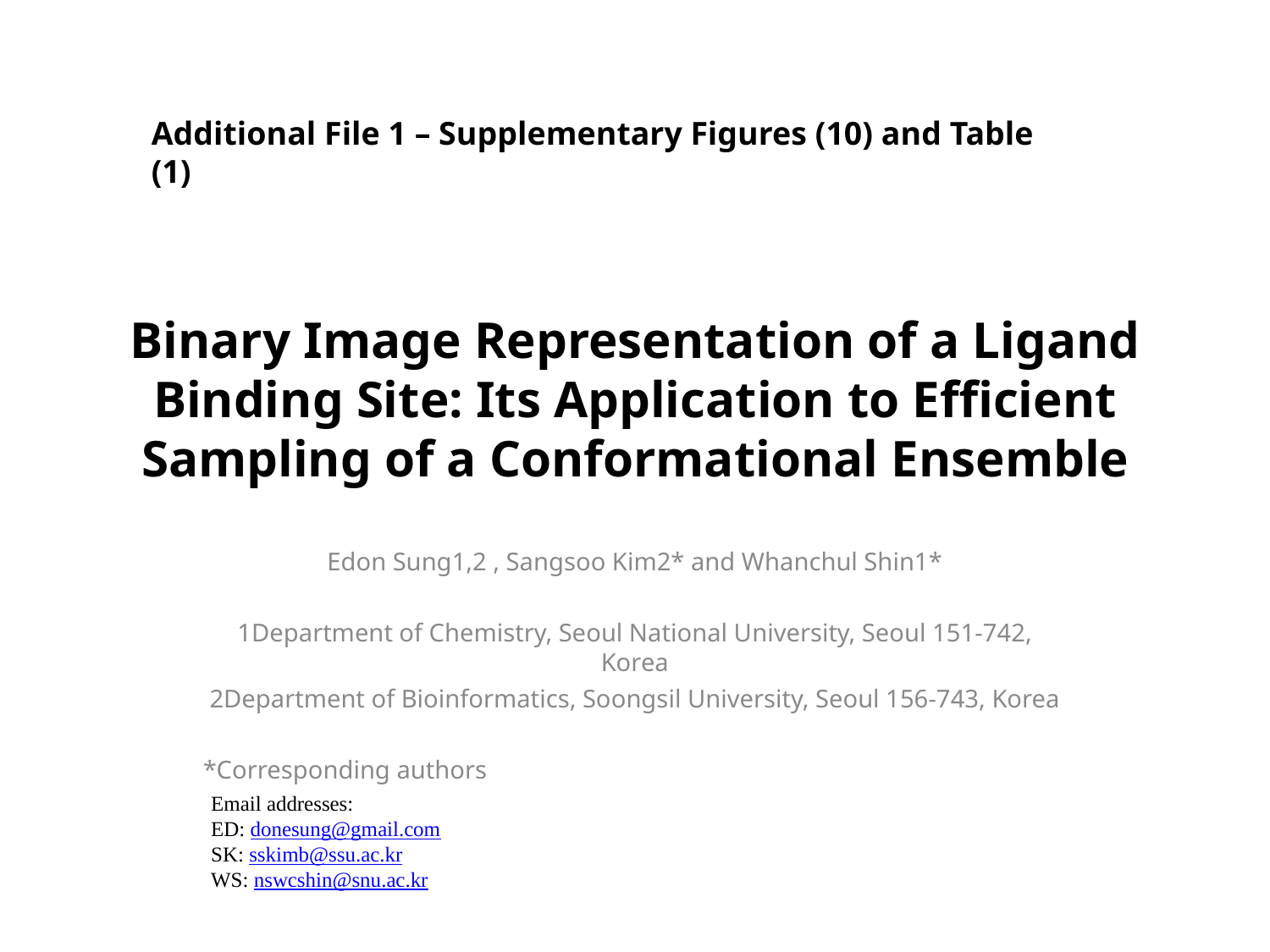

Additional File 1 – Supplementary Figures (10) and Table (1)
# Binary Image Representation of a Ligand Binding Site: Its Application to Efficient Sampling of a Conformational Ensemble
Edon Sung1,2 , Sangsoo Kim2* and Whanchul Shin1*
1Department of Chemistry, Seoul National University, Seoul 151-742, Korea
2Department of Bioinformatics, Soongsil University, Seoul 156-743, Korea
*Corresponding authors
Email addresses:
ED: donesung@gmail.com
SK: sskimb@ssu.ac.kr
WS: nswcshin@snu.ac.kr

## Slide 2
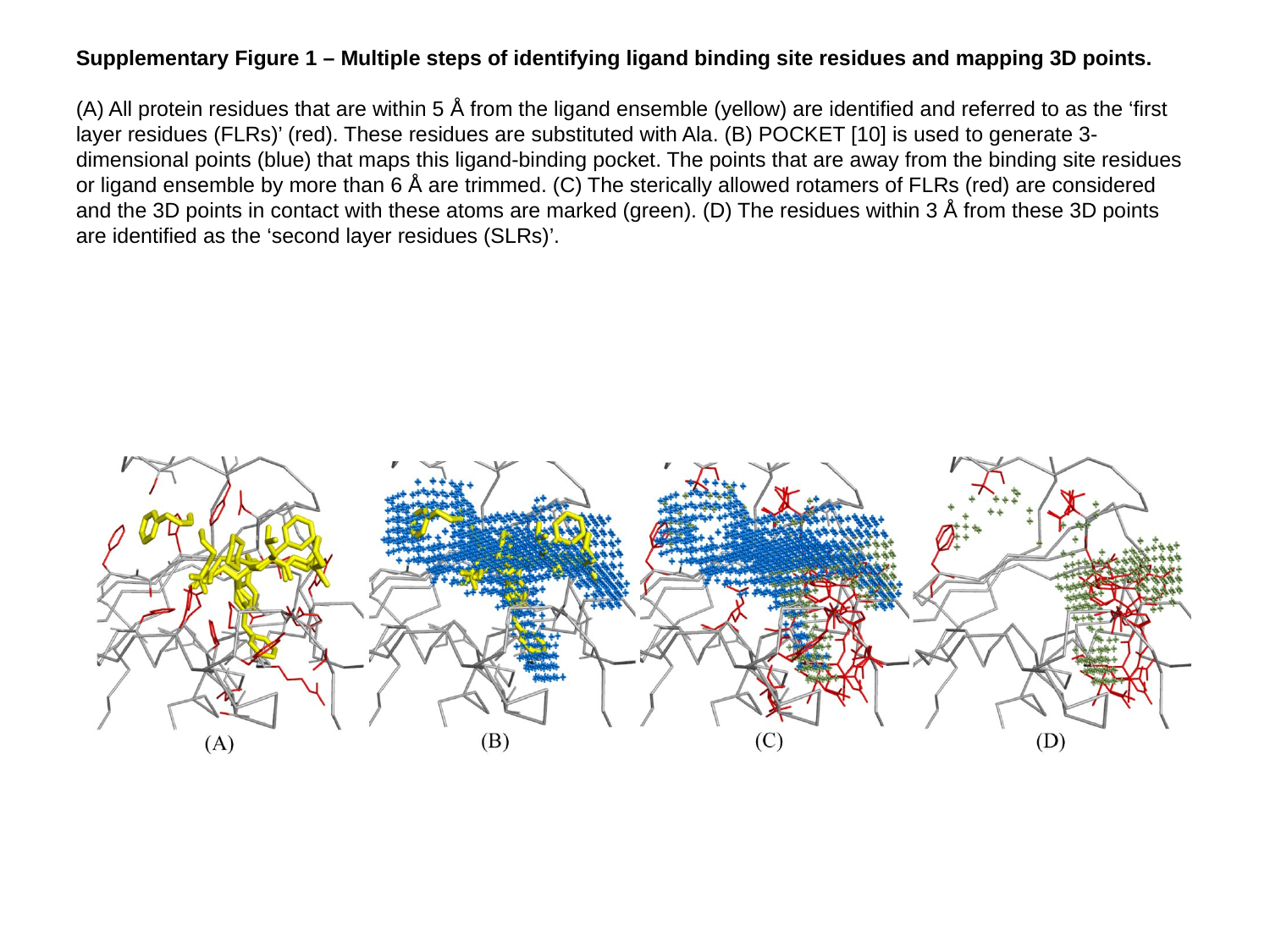

Supplementary Figure 1 – Multiple steps of identifying ligand binding site residues and mapping 3D points.
(A) All protein residues that are within 5 Å from the ligand ensemble (yellow) are identified and referred to as the ‘first layer residues (FLRs)’ (red). These residues are substituted with Ala. (B) POCKET [10] is used to generate 3-dimensional points (blue) that maps this ligand-binding pocket. The points that are away from the binding site residues or ligand ensemble by more than 6 Å are trimmed. (C) The sterically allowed rotamers of FLRs (red) are considered and the 3D points in contact with these atoms are marked (green). (D) The residues within 3 Å from these 3D points are identified as the ‘second layer residues (SLRs)’.

## Slide 3
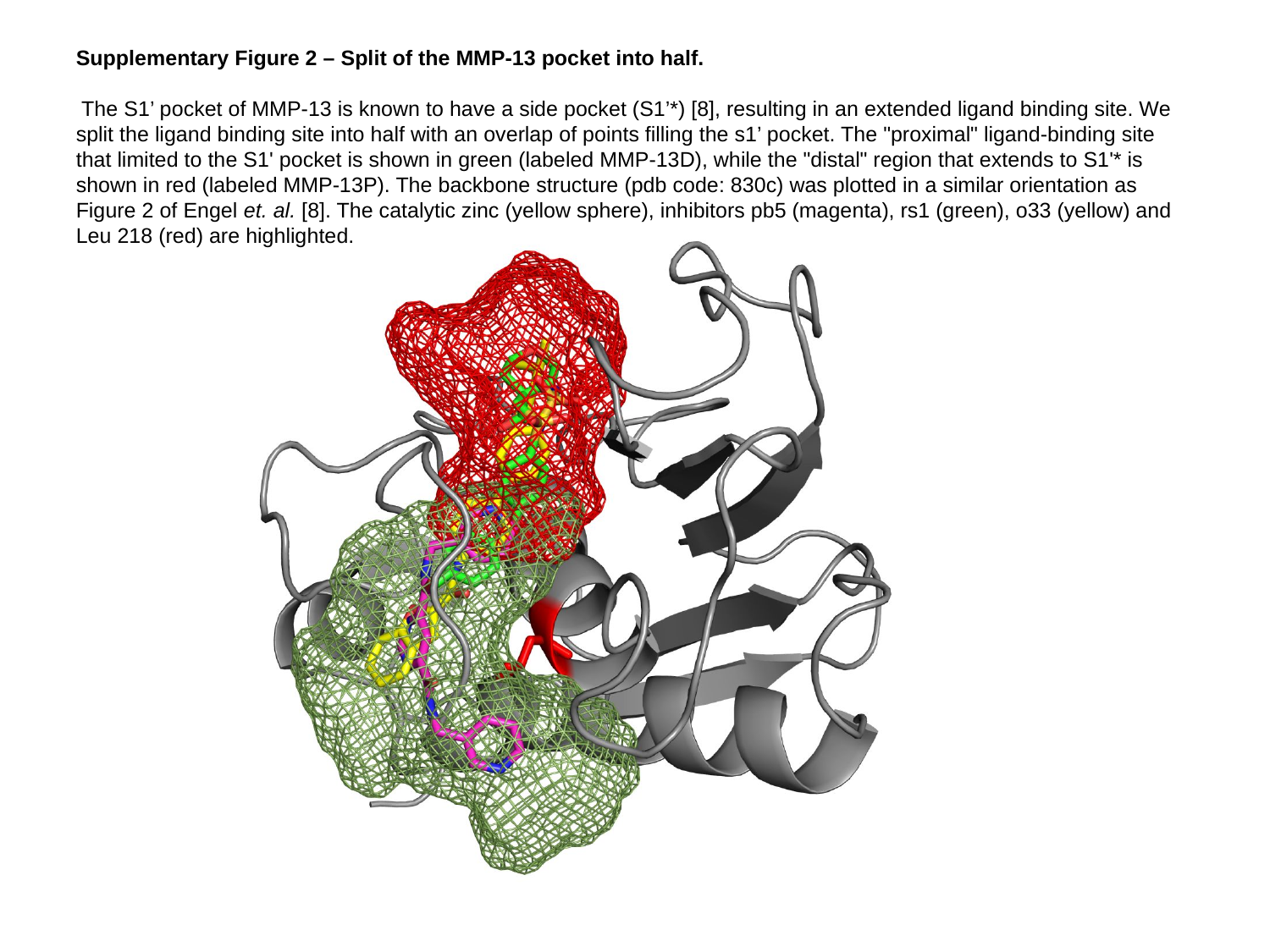

# Supplementary Figure 2 – Split of the MMP-13 pocket into half. The S1’ pocket of MMP-13 is known to have a side pocket (S1’*) [8], resulting in an extended ligand binding site. We split the ligand binding site into half with an overlap of points filling the s1’ pocket. The "proximal" ligand-binding site that limited to the S1' pocket is shown in green (labeled MMP-13D), while the "distal" region that extends to S1'* is shown in red (labeled MMP-13P). The backbone structure (pdb code: 830c) was plotted in a similar orientation as Figure 2 of Engel et. al. [8]. The catalytic zinc (yellow sphere), inhibitors pb5 (magenta), rs1 (green), o33 (yellow) and Leu 218 (red) are highlighted.

## Slide 4
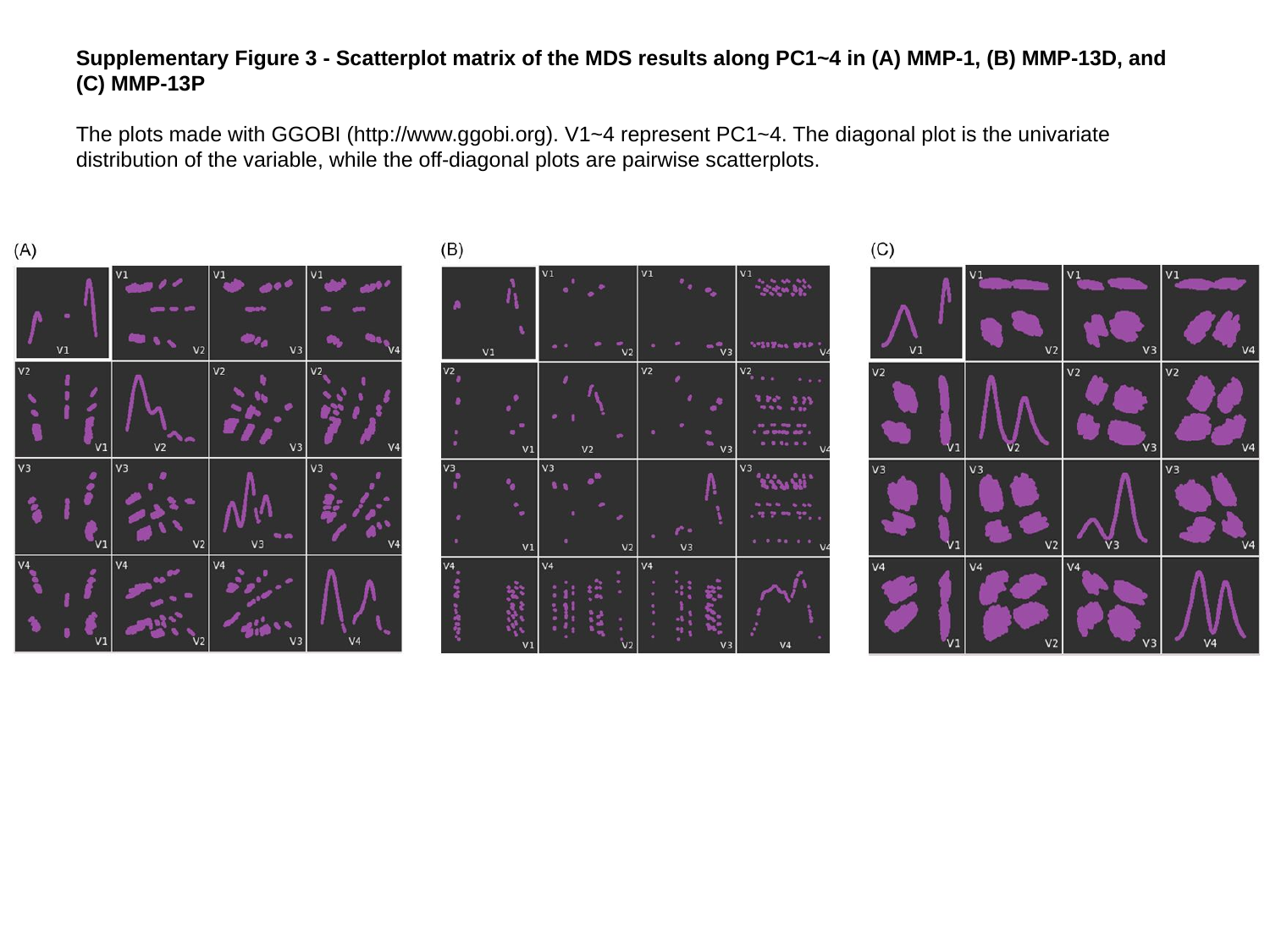

# Supplementary Figure 3 - Scatterplot matrix of the MDS results along PC1~4 in (A) MMP-1, (B) MMP-13D, and (C) MMP-13P The plots made with GGOBI (http://www.ggobi.org). V1~4 represent PC1~4. The diagonal plot is the univariate distribution of the variable, while the off-diagonal plots are pairwise scatterplots.

## Slide 5
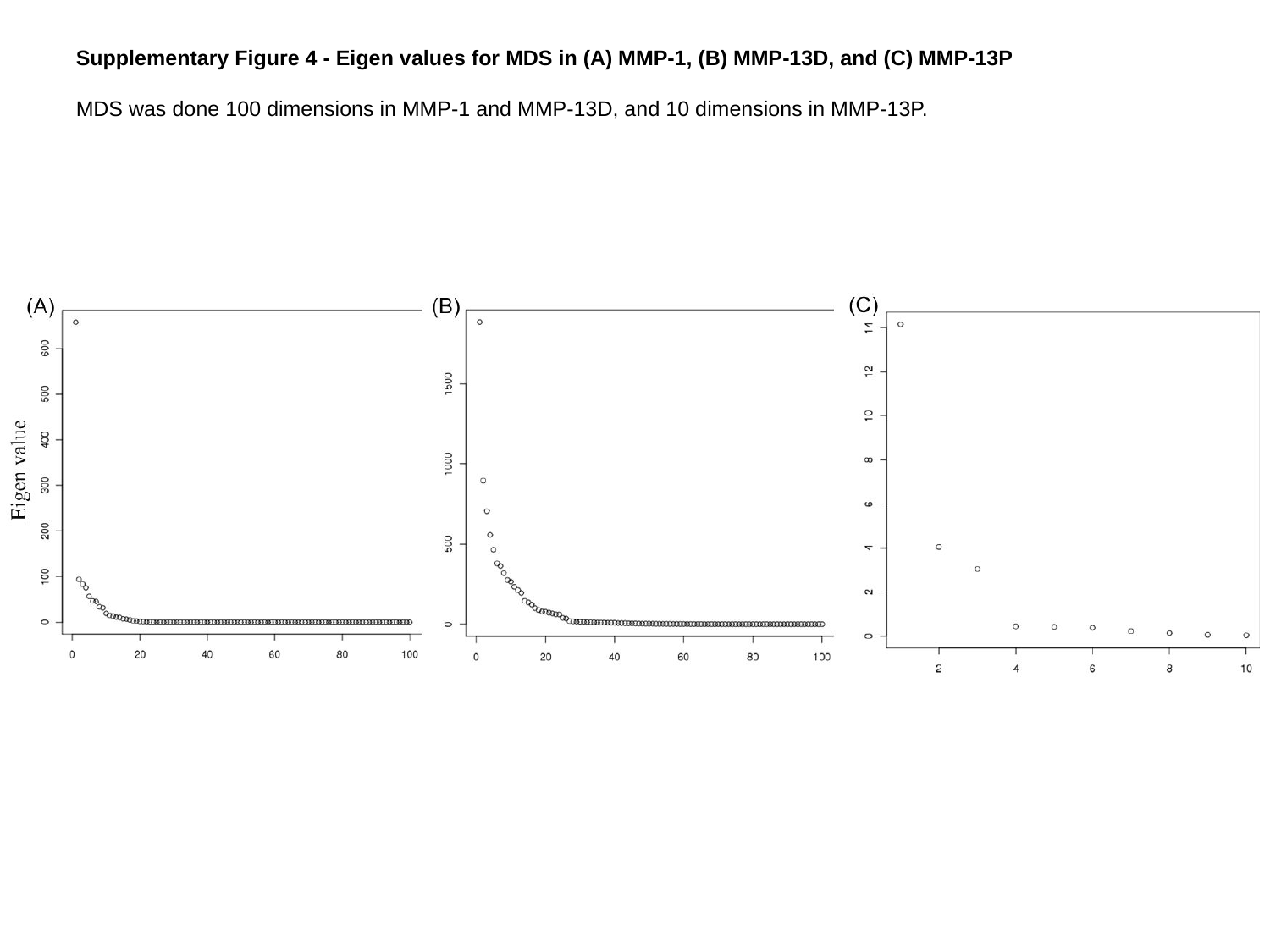

# Supplementary Figure 4 - Eigen values for MDS in (A) MMP-1, (B) MMP-13D, and (C) MMP-13P MDS was done 100 dimensions in MMP-1 and MMP-13D, and 10 dimensions in MMP-13P.

## Slide 6
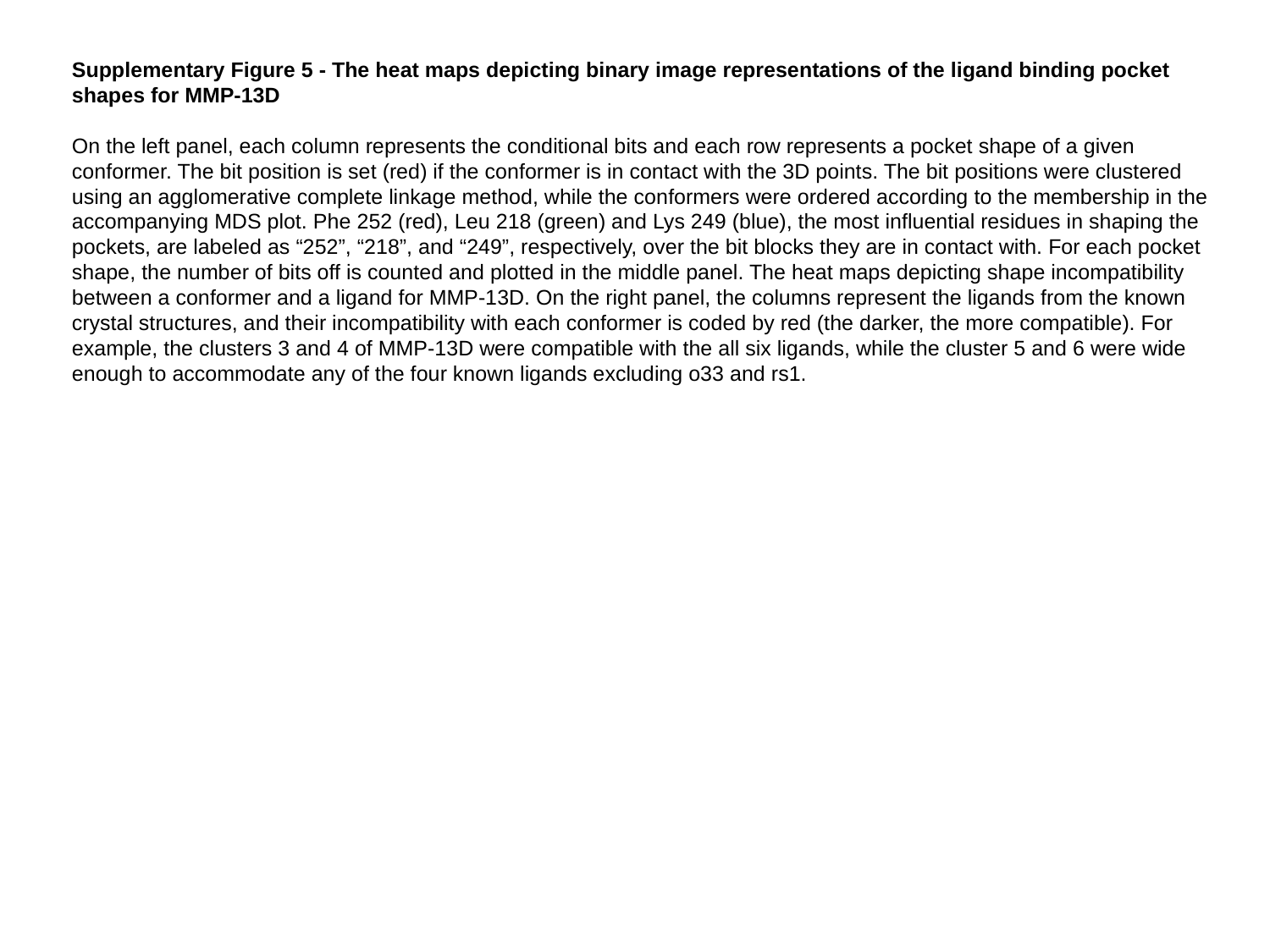

Supplementary Figure 5 - The heat maps depicting binary image representations of the ligand binding pocket shapes for MMP-13D
On the left panel, each column represents the conditional bits and each row represents a pocket shape of a given conformer. The bit position is set (red) if the conformer is in contact with the 3D points. The bit positions were clustered using an agglomerative complete linkage method, while the conformers were ordered according to the membership in the accompanying MDS plot. Phe 252 (red), Leu 218 (green) and Lys 249 (blue), the most influential residues in shaping the pockets, are labeled as “252”, “218”, and “249”, respectively, over the bit blocks they are in contact with. For each pocket shape, the number of bits off is counted and plotted in the middle panel. The heat maps depicting shape incompatibility between a conformer and a ligand for MMP-13D. On the right panel, the columns represent the ligands from the known crystal structures, and their incompatibility with each conformer is coded by red (the darker, the more compatible). For example, the clusters 3 and 4 of MMP-13D were compatible with the all six ligands, while the cluster 5 and 6 were wide enough to accommodate any of the four known ligands excluding o33 and rs1.

## Slide 7
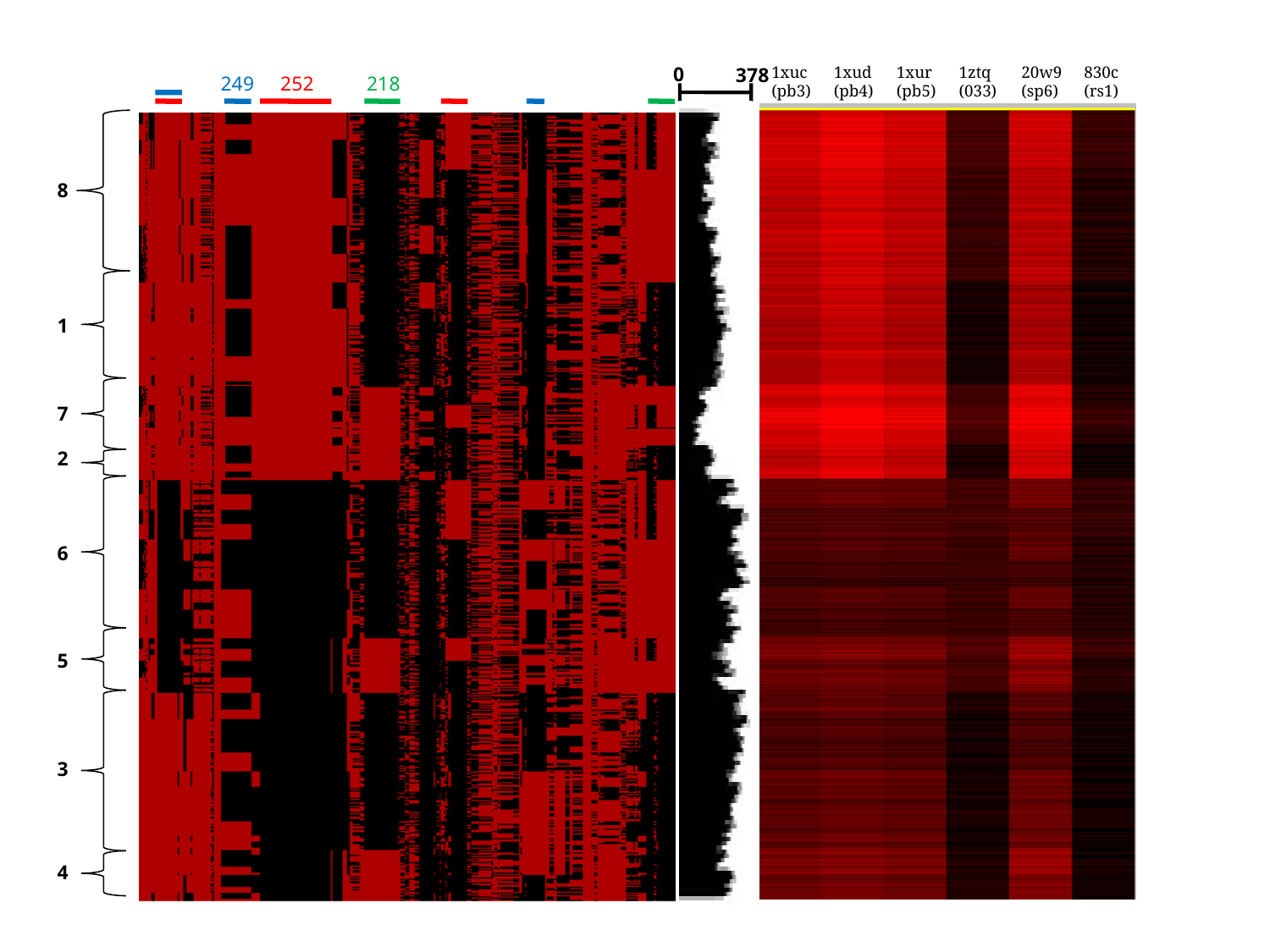

0
1xuc (pb3)
1xud (pb4)
1xur (pb5)
1ztq (033)
20w9 (sp6)
830c(rs1)
378
249
252
218
8
1
7
2
6
5
3
4

## Slide 8
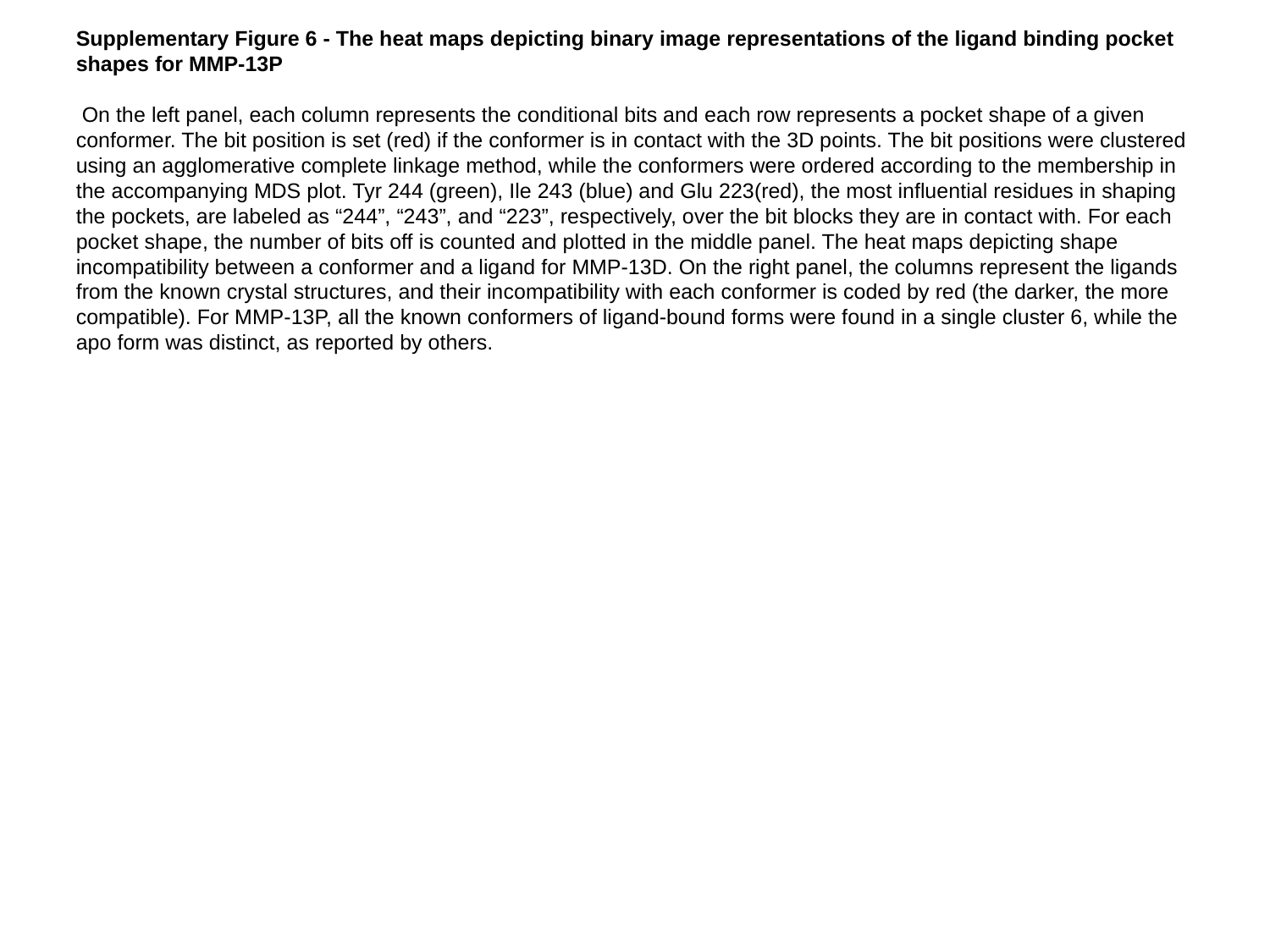

# Supplementary Figure 6 - The heat maps depicting binary image representations of the ligand binding pocket shapes for MMP-13P On the left panel, each column represents the conditional bits and each row represents a pocket shape of a given conformer. The bit position is set (red) if the conformer is in contact with the 3D points. The bit positions were clustered using an agglomerative complete linkage method, while the conformers were ordered according to the membership in the accompanying MDS plot. Tyr 244 (green), Ile 243 (blue) and Glu 223(red), the most influential residues in shaping the pockets, are labeled as “244”, “243”, and “223”, respectively, over the bit blocks they are in contact with. For each pocket shape, the number of bits off is counted and plotted in the middle panel. The heat maps depicting shape incompatibility between a conformer and a ligand for MMP-13D. On the right panel, the columns represent the ligands from the known crystal structures, and their incompatibility with each conformer is coded by red (the darker, the more compatible). For MMP-13P, all the known conformers of ligand-bound forms were found in a single cluster 6, while the apo form was distinct, as reported by others.

## Slide 9
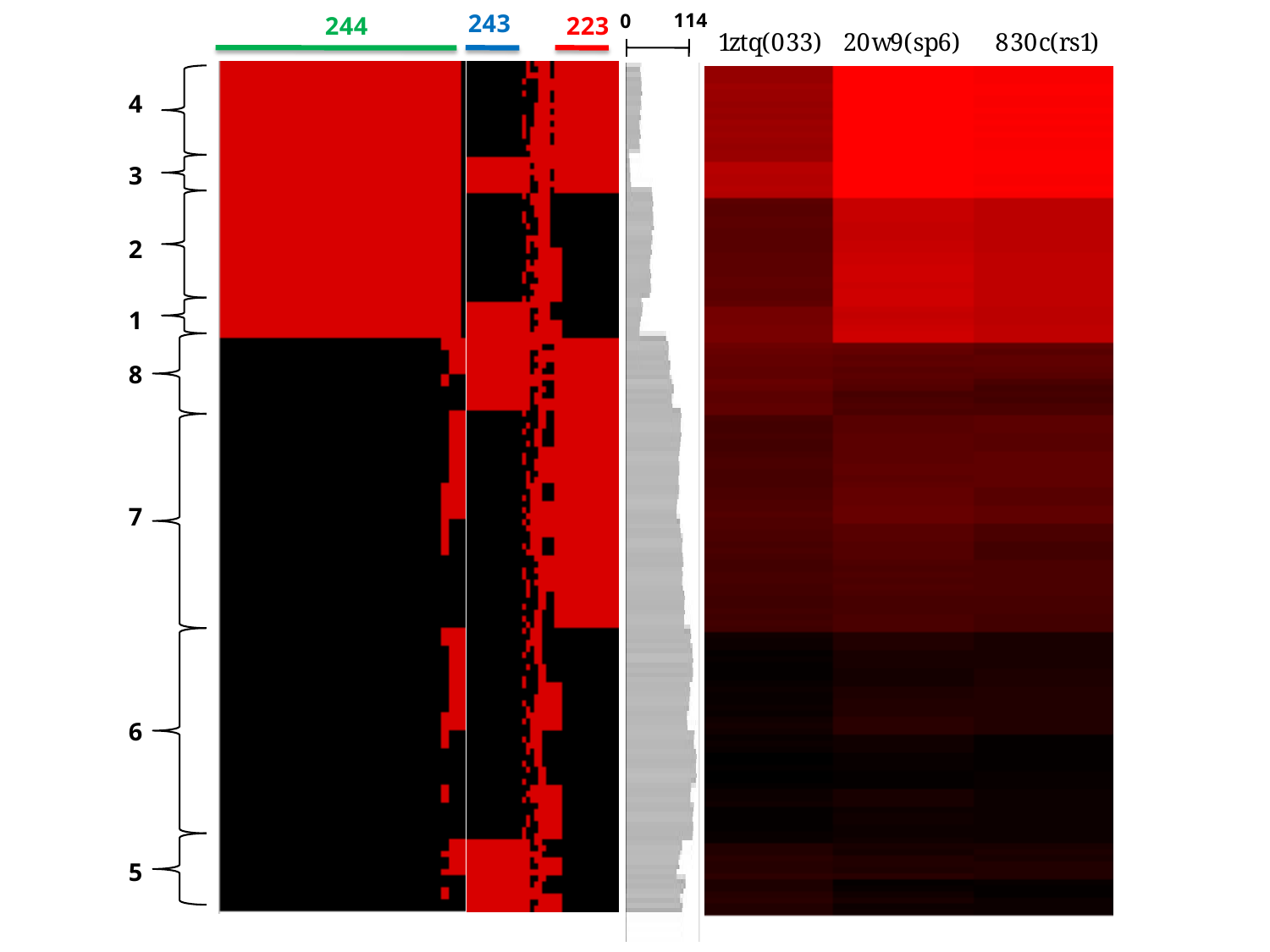

## Slide 10
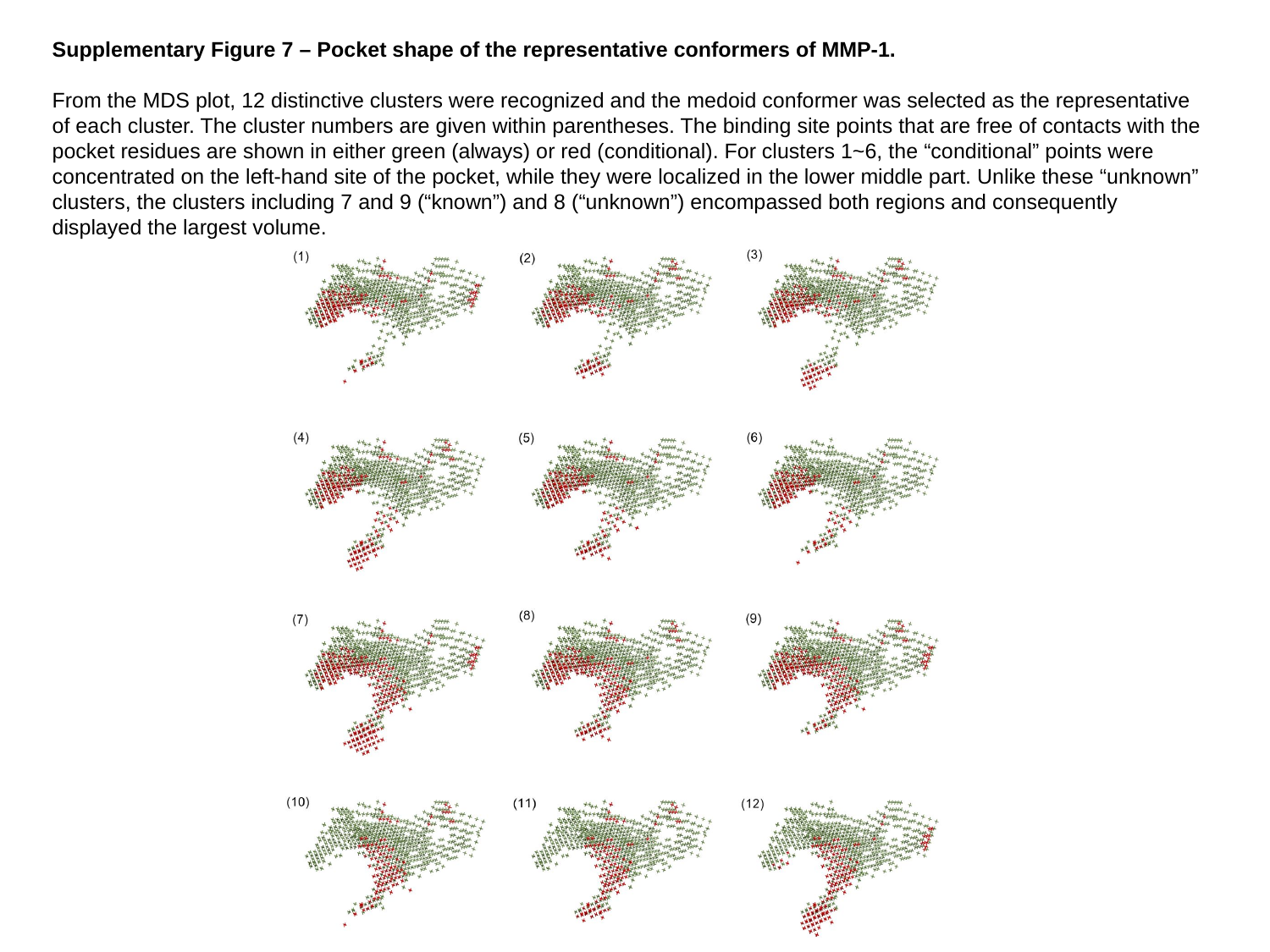

Supplementary Figure 7 – Pocket shape of the representative conformers of MMP-1.
From the MDS plot, 12 distinctive clusters were recognized and the medoid conformer was selected as the representative of each cluster. The cluster numbers are given within parentheses. The binding site points that are free of contacts with the pocket residues are shown in either green (always) or red (conditional). For clusters 1~6, the “conditional” points were concentrated on the left-hand site of the pocket, while they were localized in the lower middle part. Unlike these “unknown” clusters, the clusters including 7 and 9 (“known”) and 8 (“unknown”) encompassed both regions and consequently displayed the largest volume.

## Slide 11
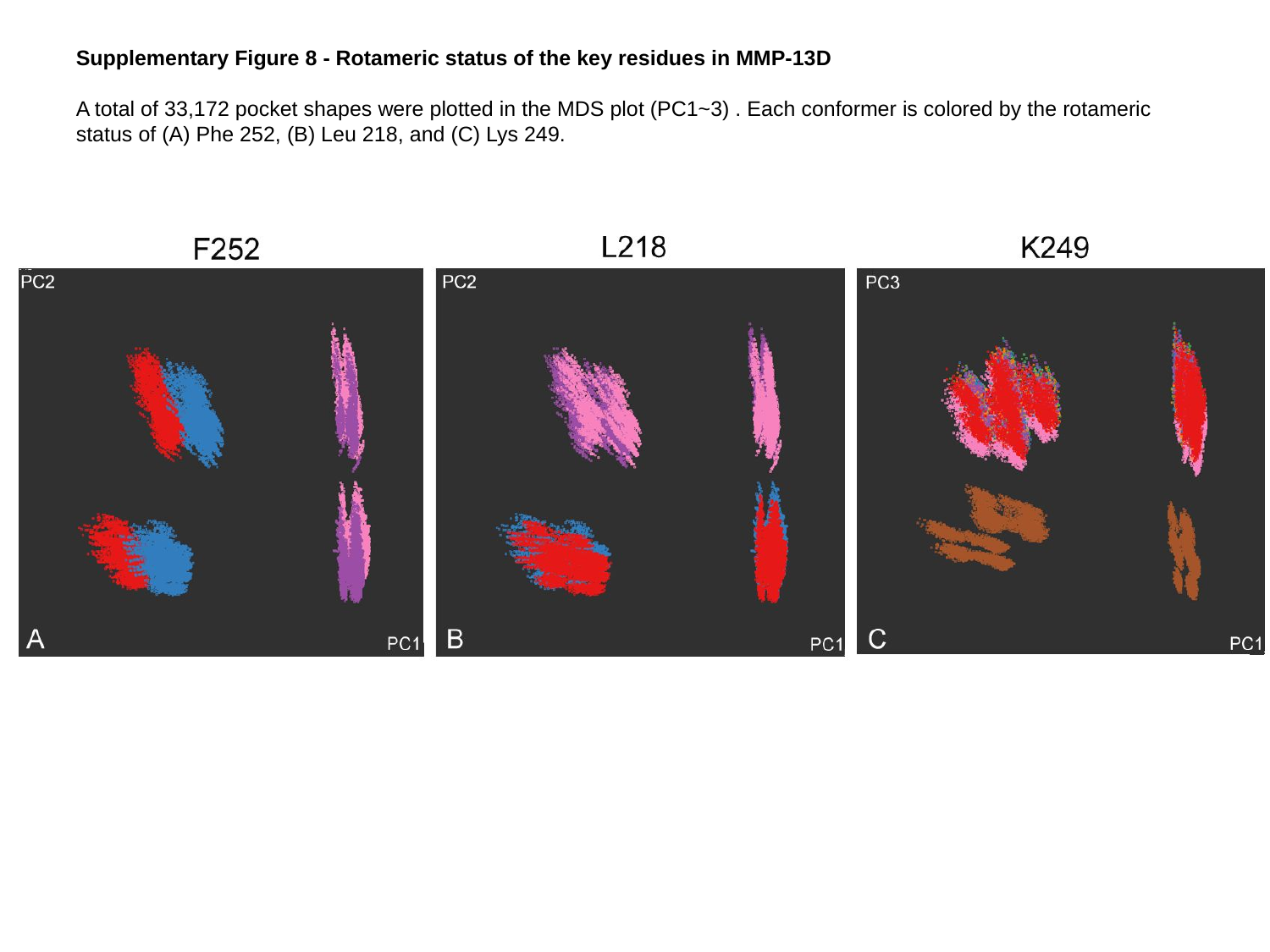

# Supplementary Figure 8 - Rotameric status of the key residues in MMP-13D A total of 33,172 pocket shapes were plotted in the MDS plot (PC1~3) . Each conformer is colored by the rotameric status of (A) Phe 252, (B) Leu 218, and (C) Lys 249.

## Slide 12
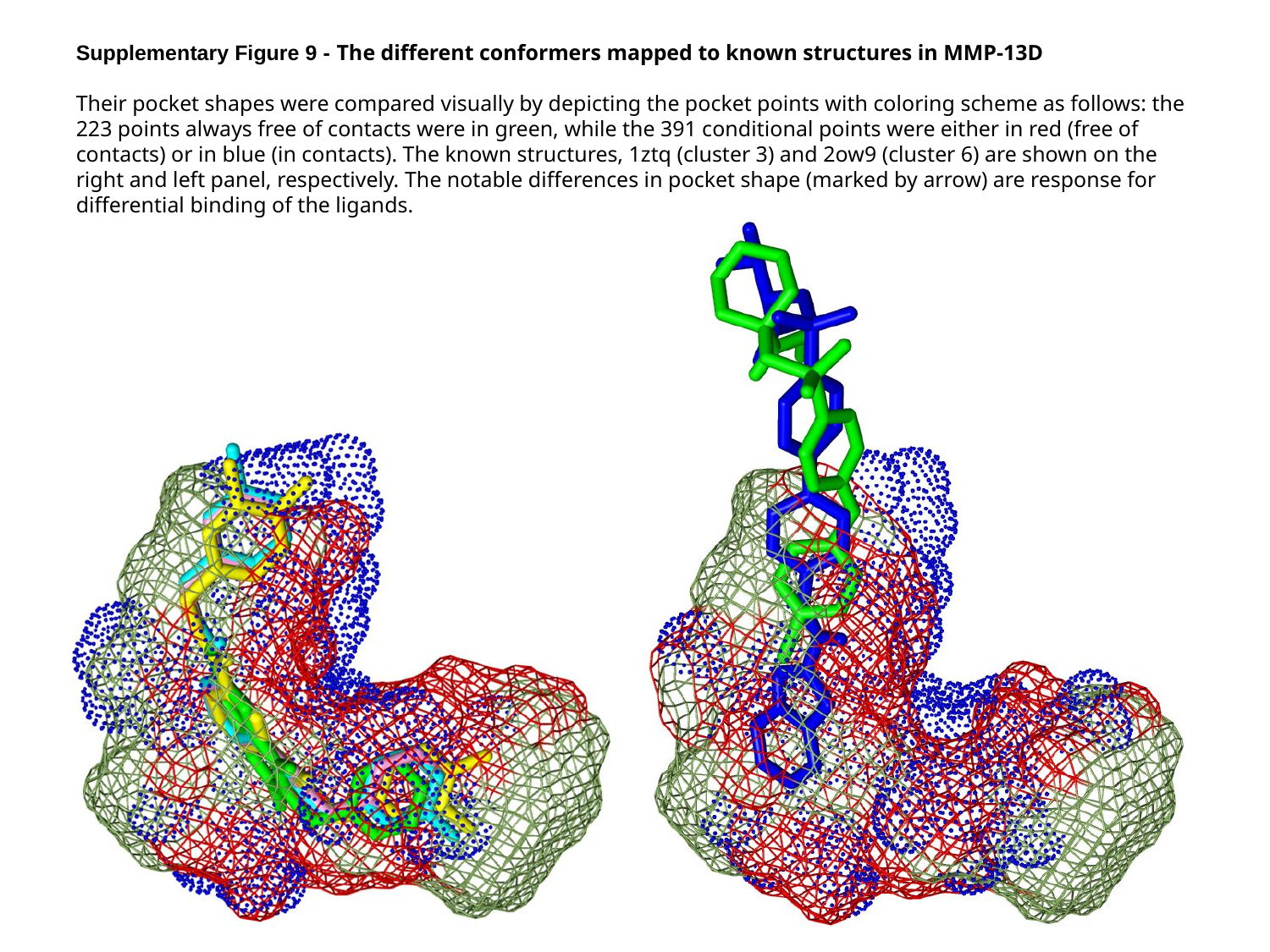

# Supplementary Figure 9 - The different conformers mapped to known structures in MMP-13D Their pocket shapes were compared visually by depicting the pocket points with coloring scheme as follows: the 223 points always free of contacts were in green, while the 391 conditional points were either in red (free of contacts) or in blue (in contacts). The known structures, 1ztq (cluster 3) and 2ow9 (cluster 6) are shown on the right and left panel, respectively. The notable differences in pocket shape (marked by arrow) are response for differential binding of the ligands.

## Slide 13
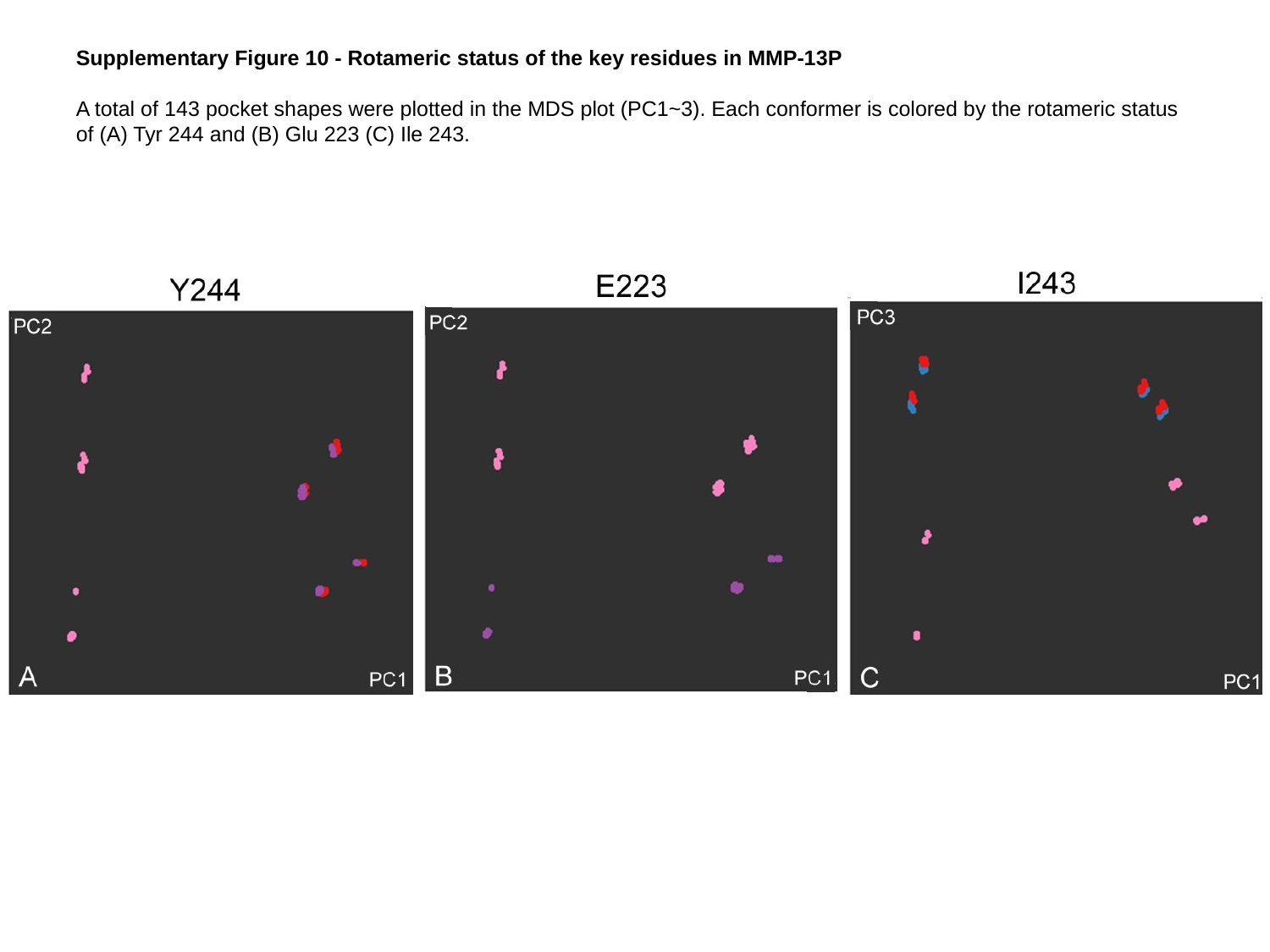

# Supplementary Figure 10 - Rotameric status of the key residues in MMP-13P A total of 143 pocket shapes were plotted in the MDS plot (PC1~3). Each conformer is colored by the rotameric status of (A) Tyr 244 and (B) Glu 223 (C) Ile 243.

## Slide 14
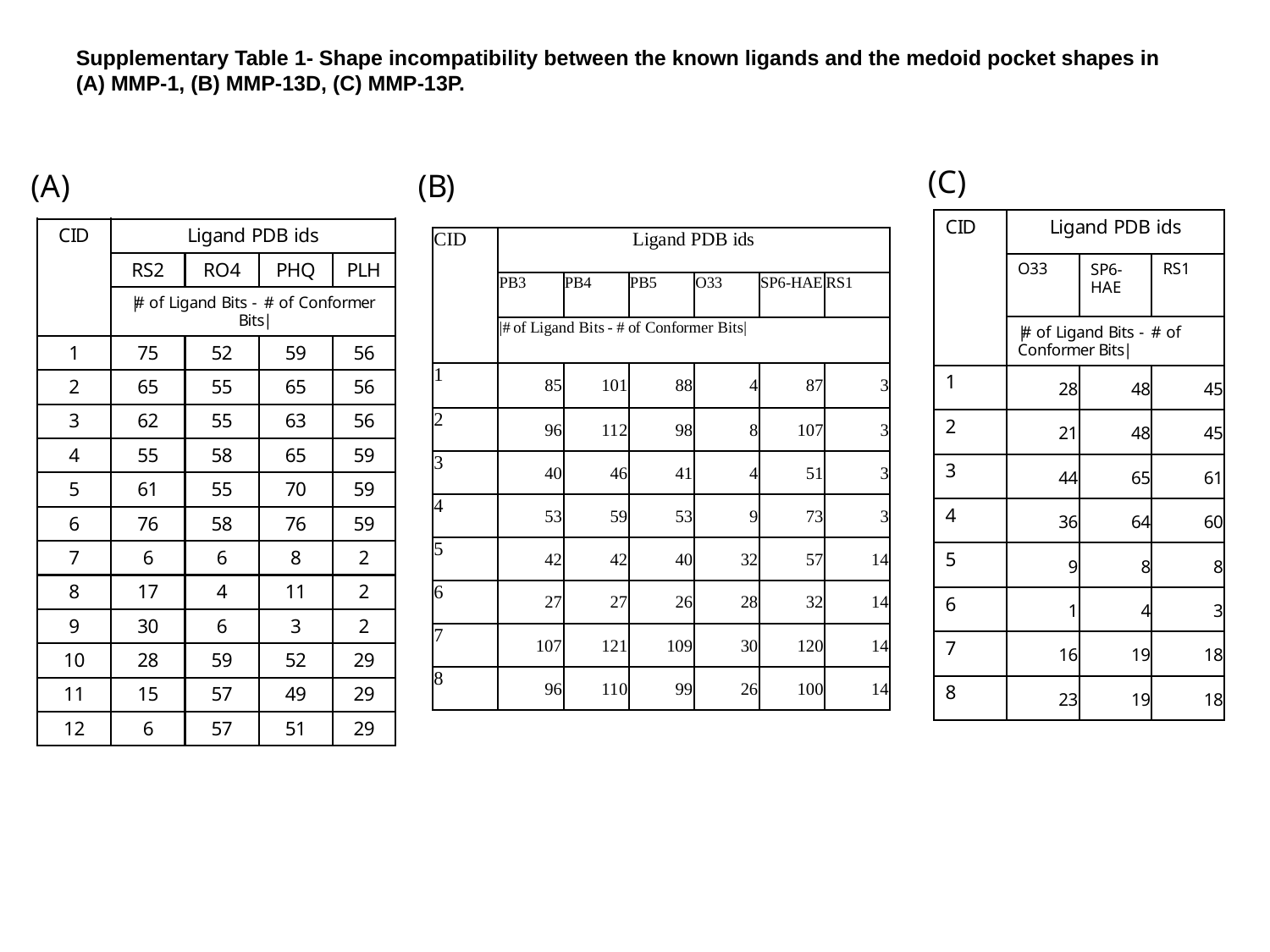

# Supplementary Table 1- Shape incompatibility between the known ligands and the medoid pocket shapes in (A) MMP-1, (B) MMP-13D, (C) MMP-13P.
