# Supplementary material for: Binary image representation of a ligand binding site: its application to efficient sampling of a conformational ensemble
Source: BMC Bioinformatics. 2010 May 18;11:256. doi: 10.1186/1471-2105-11-256 (PMC3098062; doi:10.1186/1471-2105-11-256)
Supplement: Additional File 2 — Supplementary Note. This file contains additional description of comparative features of our ROTAIMAGE and DYNASITE or Kalblad et al. [25]. [file 1471-2105-11-256-S2.DOC]

Supplementary Note

# Binary Image Representation of a Ligand Binding Site: Its Application to Efficient Sampling of a Conformational Ensemble

### Edon Sung^1,2^ , Sangsoo Kim^2^* and Whanchul Shin^1^*

^1^Department of Chemistry, Seoul National University, Seoul 151-742, Korea

^2^Department of Bioinformatics, Soongsil University, Seoul 156-743, Korea

*Corresponding authors

# Comparison of the features of ROTAIMAGE and DYNASITE

The major objectives of ROTAIMAGE are as follows:

1. The methods we propose here has to do with dealing with ligand binding site conformers that are generated by exhaustive search of rotameric combination of side chains on fixed backbone atoms
2. Our specific aim is in keeping the number of conformers minimum without losing the details too much
3. The unique feature of our method is the management of conformers using bit strings as used in image recognition techniques

One of the methods that follow similar objectives as ours is DYNASITE, which was applied to MMP-1 [1, 2]. This is a method that generates binding site conformers by exhaustive search of rotameric combination of side chains on fixed backbone atoms as ours. However, there are some differences as summarized as follows:

| **Item of MMP-1** | **DYNASITE [2]** | **ROTAIMAGE (ours)** |
| --- | --- | --- |
| Method to select ligand binding site residues | Manually defined (residues were selected based on the evidence of movement from literature) | Automatically selected based on distance criteria (see Methods for details of FLRs, SLRs, and CCRs) |
| Number of ligand binding site residues | 6 | 13 |
| Number of nonclashing conformers | 2,115 | 362,862 |
| Method to 'prune' similar conformers other than clustering | none | based on the identity of bit strings that approximate the pocket shape |
| Number of conformers used in clustering | 2,115 (+template) | 8,000 |
| Dissimilarity in clustering analysis | rmsd between conformers | pocket shape dissimilarity (Equation (2)) |
| Number of clusters | 15 | 12 |
| Method to pick up representative conformer from each cluster | the conformer with the lowest relative energy | the medoid conformer |

Both methods suggested a dozen or so multiple solutions for the ligand binding site conformers. For MMP-1 ligand binding sites, there were two kinds of the conformers reported by X-ray crystallographic studies. Those solutions from both methods included these two kinds of conformers. One may then ask, “What is the point of having another method if both perform similarly?” Since DYNASITE used only the residues whose conformational changes had been supported by structural studies in the literature, it is not surprising that it performed well in the test measuring the ability to reproduce the known conformers. Unlike DYNASITE, our ROTAIMAGE has the ability to 'prune' conformers that are similar enough to generate virtually identical pocket shapes. This allowed our method to handle many more conformers generated from the more binding site residues. Consequently we could devise a distance-based objective method to select the residues whose rotameric conformations are explored.

Of course our method also has some limitations.  The practical limit to the number of conformers handled by our method is imposed by the clustering algorithm.  Clustering analysis involving more than a few tens of thousands of bit strings or pocket shapes takes extremely long.  For example, the 'distal' S1' pocket of MMP-13 had the same number binding site residues as MMP-1, but several orders of magnitude more conformers were generated. Even after 'pruning', more than 30,000 bit strings remained and were used in the subsequent clustering analysis.

The real benefit of these explorations of conformational space is not just to reproduce the known conformers, but to identify candidates that might not be captured in the crystal structures and suggest them for docking experiments. The potential candidate does not have to be the lowest energy conformer, as the high energy conformer may be stabilized by interaction with a ligand. Both methods presented a number of solutions not observed by experiments. While some of them might be unrealistic, some others may yet to be observed. Thus it is not straightforward to compare these methods quantitatively.

# References

1. Källblad P, Dean PM: **Efficient conformational sampling of local side-chain flexibility.** *J Mol Biol* 2003, **326**:1651–65.
2. Källblad P, Todorov NP, Willems HMG, Alberts IL: **Receptor Flexibility in the in Silico Screening of Reagents in the S1’ Pocket of Human Collagenase**. *J. Med. Chem*. 2004, **47**:2761-2767.
